# MAPO-18 Catalysts for the Methanol to Olefins Process: Influence of Catalyst Acidity in a High-Pressure Syngas (CO and H_2_) Environment

**DOI:** 10.1021/acscatal.1c04694

**Published:** 2022-01-11

**Authors:** Jingxiu Xie, Daniel S. Firth, Tomás Cordero-Lanzac, Alessia Airi, Chiara Negri, Sigurd Øien-Ødegaard, Karl Petter Lillerud, Silvia Bordiga, Unni Olsbye

**Affiliations:** † Centre for Materials Science and Nanotechnology, Department of Chemistry, University of Oslo, Sem Saelandsvei 26, Oslo N-0315, Norway; ‡ Department of Chemistry, NIS and INSTM Reference Centre, Università di Torino, Via G. Quarello 15, I-10135 and Via P. Giuria 7, Torino 10125, Italy

**Keywords:** methanol-to-hydrocarbons, methanol to olefins, zeolites, SAPO, synthesis gas, alkene hydrogenation, lifetime

## Abstract

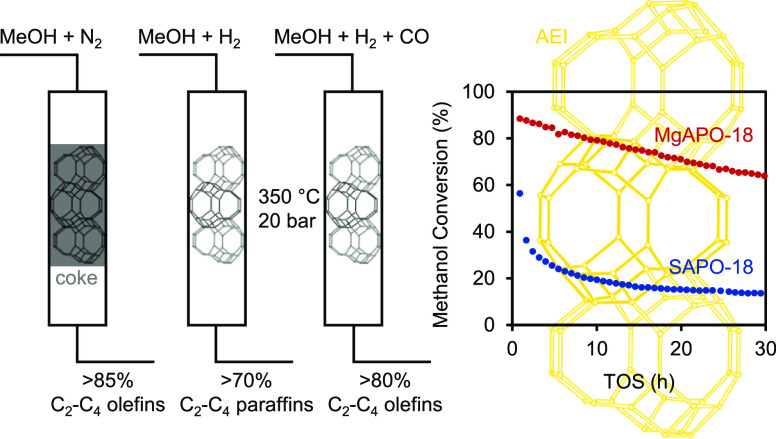

The transition from integrated petrochemical complexes toward decentralized chemical plants utilizing distributed feedstocks calls for simpler downstream unit operations. Less separation steps are attractive for future scenarios and provide an opportunity to design the next-generation catalysts, which function efficiently with effluent reactant mixtures. The methanol to olefins (MTO) reaction constitutes the second step in the conversion of CO_2_, CO, and H_2_ to light olefins. We present a series of isomorphically substituted zeotype catalysts with the AEI topology (MAPO-18s, M = Si, Mg, Co, or Zn) and demonstrate the superior performance of the M(II)-substituted MAPO-18s in the conversion of MTO when tested at 350 °C and 20 bar with reactive feed mixtures consisting of CH_3_OH/CO/CO_2_/H_2_. Co-feeding high pressure H_2_ with methanol improved the catalyst activity over time, but simultaneously led to the hydrogenation of olefins (olefin/paraffin ratio < 0.5). Co-feeding H_2_/CO/CO_2_/N_2_ mixtures with methanol revealed an important, hitherto undisclosed effect of CO in hindering the hydrogenation of olefins over the Brønsted acid sites (BAS). This effect was confirmed by dedicated ethene hydrogenation studies in the absence and presence of CO co-feed. Assisted by spectroscopic investigations, we ascribe the favorable performance of M(II)APO-18 under co-feed conditions to the importance of the M(II) heteroatom in altering the polarity of the M–O bond, leading to stronger BAS. Comparing SAPO-18 and MgAPO-18 with BAS concentrations ranging between 0.2 and 0.4 mmol/g_cat_, the strength of the acidic site and not the density was found to be the main activity descriptor. MgAPO-18 yielded the highest activity and stability upon syngas co-feeding with methanol, demonstrating its potential to be a next-generation MTO catalyst.

## Introduction

The conversion of methanol to olefins (MTO) using zeolite/zeotype catalysts provides a viable key step to the production of chemicals from alternative carbon raw materials, including natural gas, CO_2_, biomass, and municipal waste.^1,2^ The industrial process typically operates at 350–500 °C and 1 bar, using SAPO-34 (12-/8-ring CHA topology) and ZSM-5 (10-ring MFI topology) catalysts.^3−5^ High selectivity toward C_2_–C_4_ olefins achieved with SAPO-34 is due to its topology which limits product effusion to molecules smaller than 3.8 Å.^6^ On the other hand, high selectivity toward propene is attained with ZSM-5 by the recycling of products and operation at higher temperatures (>500 °C) to facilitate the cracking of the hydrocarbon products.^3^ On the molecular scale, MTO product distribution is governed by a delicate balance between relative diffusivities, competitive adsorption, and reaction on the internal surface of the microporous catalysts.^7^ The reaction path is dominated by the dual-cycle mechanism (Figure S1), in which alkenes and arenes [the hydrocarbon (HC) pool species] are methylated and subsequently cracked or dealkylated to form light olefins.^3,8−10^ The cycles are connected through hydrogen transfer reactions, that are also core to HC pool initiation by first C–C bond formation, as well as deactivation by coke formation.^1,11−13^ In addition to the zeolite/zeotype topology,^14−17^ the number/strength/distribution of acidic sites,^18−21^ lattice defects, and crystal size/morphology influence MTO catalyst performance.^22−24^ Reaction conditions such as temperature, methanol partial pressure, and contact time^25,26^ are also paramount to optimal catalyst performance.

Recent breakthroughs on the MTO process focuses on high reaction pressures, for example >20 bar with H_2_ co-feeding, due to the development of tandem processes to directly convert CO/CO_2_/H_2_ feed to lower olefins *via* methanol.^27−31^ Bhan and co-workers demonstrated that co-feeding H_2_ at high pressures (400 °C, 4–30 bar *P*_H2_, and 0.13 bar *P*_MeOH_) over SAPO-34 mitigated catalyst deactivation without significantly decreasing selectivity toward lower olefins.^32^ They extended this strategy to other zeolites with CHA, AEI, FER, and BEA topology and isostructural SAPO-34 and SSZ-13.^33,34^ Upon H_2_ co-feeding, the more acidic SSZ-13 not only had a longer lifetime than SAPO-34 but also higher selectivities toward methane and paraffins. The hydrogenation of coke precursors including formaldehyde and 1,3-butadiene was concluded to suppress the production of deactivation-inducing polycyclics thereby improving the catalyst lifetime. Independently, the group of Liu arrived at a similar strategy to prolong the lifetime of SAPO-34 (425 °C, 40 bar, and H_2_/MeOH/H_2_O = 6/1/5.33) and highlighted the synergistic effect of co-feeding H_2_ and H_2_O at high pressures.^35^ They showed that co-feeding H_2_ with methanol led to a longer lifetime but an obvious decrease in selectivity toward lower olefins. Hence, co-feeding H_2_ and H_2_O was required to improve the stability and maintain olefins selectivity. Unfortunately, high pressure steam addition simultaneously led to SAPO-34 structural damage.^35^ Clearly, challenges remain in bridging the gaps between the current and future MTO process conditions.

In this contribution, we study zeotype acidity in the presence of high CO/CO_2_/H_2_/N_2_ co-feed partial pressures, for a next-generation MTO process. We focus on the AEI topology due to its high propene selectivity^17^ and vary acidity by heteroatom M substitution of Si, Mg, Co, and Zn and by M/Al elemental ratio.^36−38^ SAPO-18 expectedly outperformed the M(II)APO-18s under typical MTO conditions of 350 °C and 1 bar, methanol in N_2_ feed, but the M(II)APO-18s achieved 2–3 times higher propene yield than SAPO-18 at 350 °C, 20 bar, and methanol in syngas feed. The superior catalytic stability of the more acidic MgAPO-18s [Brønsted acid sites, (BAS) = 0.2 to 0.4 mmol/g_cat_] originated from the acid site strength and not density, thus revealing their potential as next-generation MTO catalysts. We also emphasize the role of CO co-feeding. Beyond prior studies, which showed the role of CO in direct C–C bond formation in MTO,^39−44^ CO is here proven to prevent the hydrogenation of lower olefins.

## Experimental Methods

### Synthesis of MAPO-18 Catalysts

All catalysts were prepared *via* hydrothermal synthesis using the same organic structure-directing agent, *N*,*N*-diisopropylethylamine (DIPEA, ≥99%, Sigma-Aldrich).^45^ The other chemicals were alumina [AlO(OH), Pural, Sasol], orthophosphoric acid (85 % wt. H_3_PO_4_ in H_2_O, Sigma-Aldrich), colloidal silica (40 % wt. SiO_2_ suspension in H_2_O, Ludox AS-40, Sigma-Aldrich), magnesium acetate tetrahydrate [(CH_3_COO)_2_Mg·4H_2_O, ≥98%, Sigma-Aldrich], cobalt(II) acetate tetrahydrate [(CH_3_COO)_2_Co·4H_2_O, ≥98%, Sigma-Aldrich], zinc acetate dihydrate [(CH_3_COO)_2_Zn·2H_2_O, ≥98%, Sigma-Aldrich] and deionized water. SAPO-18 was synthesized with a gel composition of AlO(OH)/SiO_2_/H_3_PO_4_/DIPEA/H_2_O = 1/0.1/0.9/0.95/9.5 as described previously and is referred to as SAPO-18.^23^ SAPO-18a-d was synthesized by varying Si/T atomic composition in the synthesis gels. The P source, H_2_O, and DIPEA were first mixed together. Pural was subsequently added slowly with stirring for 5 min, and Ludox was finally added. The synthesis gel was left to stir for 20 min to ensure homogeneity. The gel was transferred to a Teflon-lined stainless-steel autoclave and heated at 190 °C under rotation for 12 h. MAPO-18 (where M refers to Mg, Co, or Zn) catalysts were synthesized with identical gel compositions of AlO(OH)/(CH_3_COO)_2_M/H_3_PO_4_/DIPEA/H_2_O = 1/0.1/0.9/0.95/19. MgAPO-18a-c catalysts were prepared with the same M/T atomic composition in the synthesis gels as SAPO-18a-c. The metal acetate precursor was first dissolved in minimal amount of H_2_O. The P source, H_2_O, and DIPEA were then mixed together. Pural was subsequently added slowly with stirring for 5 min, and M acetate precursor solution was finally added. The synthesis gel was left to stir for 20 min to ensure homogeneity. The gel was transferred to a Teflon-lined stainless-steel autoclave (∼50% filled) and heated at 160 °C under rotation for 8 days. All products were washed and centrifuged three times with deionized water and dried at 100 °C for 18 h. Calcination was performed at 550 °C (3 °C/min) under static air condition for 4 h.

### Catalyst Characterization

The powder X-ray diffraction (PXRD) patterns of the as-synthesized and calcined catalysts were measured using a Siemens Bruker D8 Discover instrument with Bragg–Brentano geometry by using Cu K_α_ radiation (λ = 1.5406 Å). Samples were mounted on flat sample holders and measured in the reflectance mode with Bragg–Brentano geometry. All patterns were fitted using TOPAS6. Rietveld fittings were performed using previously published crystal structures of AlPO-18, both containing OSDA and calcined catalysts.^46^ Peaks belonging to CHA impurities, both CHA intergrowths and separate phases, were fitted qualitatively by the Pawley method. Due to the high degree of disorder in the materials, quantification of these intergrowths was not attempted. The size and morphology of the calcined zeotype particles were analyzed by scanning electron microscopy (SEM), recorded with a Hitachi SU 8230 field emission scanning electron microscope. The elemental composition was determined utilizing energy-dispersive X-ray spectroscopy (EDS) attached to the same instrument. N_2_ physisorption was carried out at 77 K by using a BELSORP-mini II equipment to determine the Brunauer–Emmett–Teller (BET) surface areas and pore volumes. Calcined catalysts were outgassed under vacuum for 4 h, 0.5 h at 80 °C, followed by a period of 3 h at 300 °C. The BET surface areas were determined on the basis of a linear fit of the data in the relative pressure (*p*/*p*_0_) range of 0.005–0.05. Temperature-programmed desorption of *n*-propylamine was performed at atmospheric pressure in a fixed-bed glass reactor (inner diameter, 11 mm), similar to the procedure described previously.^47^ Calcined catalysts (250–420 μm) were pretreated at 550 °C under flowing air condition. The catalyst was then cooled to 130 °C, after which 80 mL/min N_2_ bubbled through a saturator containing *n*-propylamine at room temperature was then fed to the catalyst for 30 min. The excess amount of *n*-propylamine was removed by flowing 80 mL/min N_2_ for 4 h at 130 °C. The temperature was then increased to 550 °C (20 °C/min) and the amount of propene desorbed was quantified by using an online Pfeiffer Omnistar quadrupole mass spectrometer. Fourier-transform infrared (FTIR) spectroscopy was performed in the transmission mode using a Bruker Vertex 70 spectrophotometer (resolution: 2 cm^–1^; cryodetector: MCT) collecting 32 scans for each spectrum. An aliquot of each MAPO-18 catalyst was pressed in a self-supporting pellet enveloped in a pure gold cover and placed in a home-made quartz cell equipped with KBr windows designed to carry out spectroscopic measurements at *ca.* 77 K. The cell was connected to a vacuum line (residual pressure: 1 × 10^–4^ mbar) allowing for the thermal treatment and adsorption experiments to be carried out *in situ*. All the investigated samples were activated under dynamic vacuum at 400 °C for 90 min before each experiment. CO was dosed *in situ* and then adsorbed by cooling the system to 77 K using liquid N_2_.

### Catalytic Tests at 1 bar

The ambient pressure MTH test rig was described previously.^36^ The quantity of calcined catalyst loaded (250–420 μm) was varied depending on the reaction temperature. 100 mg of MAPO-18 was loaded in a fixed-bed U-shaped quartz reactor and heated to 550 °C (5 °C/min) in synthetic air feed (N_2_/O_2_ = 80/20 %v and 25 mL/min). At 550 °C, the synthetic air feed was switched to 100 % v O_2_ feed for 1 h, after which the temperature was decreased to the reaction temperature of 350 °C (2 °C/min) in 100 % v N_2_ feed. During the reaction, methanol was fed to the reactor by bubbling He through a saturator at 20 °C, resulting in a methanol partial pressure of 0.13 bar and WHSV of 4 g_MeOH_ g_cat_^–1^ h^–1^. The total feed flow was 40 mL/min. The effluent from the reactor was analyzed using an online gas chromatography–mass spectrometry (GC–MS) instrument (Agilent 7890 with flame ionization detector and 5975C MS detector) equipped with two Restek Rtx-DHA-150 columns. Hydrogen (Praxair, purity 6.0) was used as the carrier gas. Both methanol and dimethyl ether were considered to be reactants when calculating the conversion for activity. Product selectivity was determined based on carbon atoms measured by the FID detector.

### Catalytic Tests at 20 bar

Methanol conversion over the MAPO-18s at 20 bar in various reactive feeds was investigated using a commercial Microactivity Effi test rig from PID Eng & Tech. Blank reactor tests were also performed and they showed no reactivity of methanol or CO/CO_2_. 400 mg of calcined MAPO-18 (250–420 μm) was loaded in a silicon-coated (Silcolloy coating from SilcoTek) stainless-steel reactor with an inner diameter of 6 mm. The catalyst bed (isothermal zone of 5 cm) was supported by glass wool placed above 5 mm glass beads, and a thermocouple (Type K) was inserted in the catalyst bed. The catalyst was heated to the reaction temperature of 350 °C (5 °C/min) at 1 bar in 100 % v inert feed (N_2_ and Ar) for 1 h. No significant differences in performance at typical MTO conditions were observed with the two activation protocols (350 °C in inert atmosphere *vs* 550 °C in air). The feed flow was then switched to bypass the reactor for 4 h so as to obtain a stable methanol feed flow. N_2_ was used to pressurize the methanol liquid feed tank and line, and methanol liquid feed flow was controlled with a CORI-FLOW controller (Bronkhorst). Methanol was evaporated in the hot box at 140 °C and swept by the flowing gas stream. Methanol feed flow was 1 g/h, and internal standard Ar feed flow was 7 mLn/min. Individual gas mass flow controllers (Bronkhorst) were used to set the flow rate for each gas, namely CO_2_, CO, H_2_, N_2_, and Ar, and the gases were mixed before the methanol feed line. Total feed flow was 220 to 230 mLn/min, resulting in a GHSV of 16 000 h^–1^. The reaction pressure of 20 bar within the reactor was controlled by a back pressure regulator and this is a PID Eng & Tech patented system based on a high-speed precision servo-controlled valve (VMM01) with eight turns of rotational movement. The product stream was connected to the vent and the online gas chromatograph (Scion 456-GC). The gas chromatograph was equipped with 1 TCD and 2 FID detectors and six columns (MolSieve 13X, HayeSep Q, HayeSep N, Rt-Stabilwax, Rt-Alumina/MAPD, and Rtx-1). Helium was used as the carrier gas.

The same equipment was used for ethene hydrogenation control experiments performed at 10 bar under N_2_ and CO flows. Blank tests were also carried out and, in these cases, small conversions of *ca.* 4.5 and 2.3%, respectively, were observed. This was caused by the thermal reaction over steel tubing, reactor, and inner lines of the gas chromatograph. These values were subtracted from the shown results that only represent hydrogenation due to the acidic sites of the zeotype. 320 mg of SAPO-18 was loaded in the same reactor and subjected to the same pretreatment at 350 °C in inert atmosphere for 1 h. The reactor was then pressurized whereas the reactants mix (now 205 mLn/min, aiming at 0.25 bar C_2_ and H_2_/N_2_, or CO ratio of 3) was flowed through the bypass line for stabilization. The experiments were performed at low C_2_ partial pressure and GHSV of 18 500 h^–1^ to avoid oligomerization reactions. The carbon balance was closed at 99.5% with ethene and ethane, suggesting that the only reaction taking place was hydrogenation.

### Computational Details

Structures were optimized and properties calculated with the program Dmol3 as implemented in BIOVIA Materials Studio 2020. The calculations were done on periodic models based on one unit cell of the AlPO-18 structure with either one phosphorous replaced by a silicon or one aluminum replaced by magnesium. The unit cell composition is MgAl_23_P_24_O_96_H or SiAl_24_P_23_O_96_H and the adsorbed molecule C_2_H_4_ comes in addition. The optimizations were first calculated with the simple LDA-PWC functional with coarse convergence criteria. The optimized geometries were then optimized further with the m-GGA-M06-L functional with medium convergence criteria.^48,49^ Electron density and electrostatics were calculated and are the basis for the electron isosurface with charge distribution visualized by spectral colors, blue negative and red positive. The isosurface value used for Figure 5c,d is 0.2. Coordinates for the optimized geometries in Figure 3 are reported in Supporting Information, Section 4.

## Results and Discussion

### Catalysts

**Figure 1:**
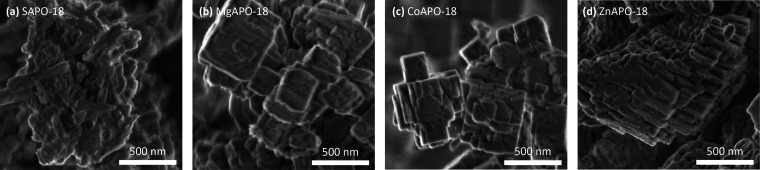
SEM images of calcined MAPO-18s. (a) SAPO-18, (b) MgAPO-18, (c) CoAPO-18, and (d) ZnAPO-18.

The MAPO-18s were prepared *via* hydrothermal synthesis using DIPEA as the structure-directing agent.^45^ Crystal sizes and morphologies of the calcined zeotypes were determined using SEM (Figure 1, Table 1). The crystals of MgAPO-18 and CoAPO-18 were cubic with rough surfaces, suggesting they are agglomerates of smaller crystals. ZnAPO-18 crystals also showed rough surfaces, but the crystals were significantly larger and not cubic. SAPO-18 was formed as rods, in agreement with the prior literature.^23^ The homogeneity of the crystals was reflected in the SEM images with lower magnification (Figure S2), with the elemental composition checked using SEM–EDS at different locations (Table S1). PXRD patterns of the as-synthesized and calcined zeotypes were complicated by the intentionally small domain size, yet confirmed the AEI topology of the calcined samples (Figure S3). One sample, ZnAPO-18, partially collapsed and contained dense phases in addition to the AEI phase after calcination. More details are reported in Supporting Information, Section S2. N_2_ adsorption–desorption isotherms are reported in Figure S4 and specific surface areas and micropore volumes are reported in Table 1. The porosity of the samples were in accordance with the XRD data, indicating highly crystalline AEI for Mg-, Co-, and Si-APO-18, whereas ZnAPO-18 contained mainly dense phases.

**Table 1:** Textural and Acidic Properties of the MAPO-18 Catalysts

						elemental composition^c^				
	crystal shape^a^	crystal size (μm)^a^	*S*_BET_ (m^2^/g)^b^	*V*_micr_ (cm^3^/g)^b^	*S*_ext_ (m^2^/g)	P/Al	M/Al	M/(Al + P)	density of *M* (mmol/g_cat_)^c^	Brønsted acidity (mmol/g_cat_)^d^
SAPO-18	rods	∼0.5	750	0.26	86	1.0	0.12	0.06	0.61	0.29
MgAPO-18	cubes	∼0.5	730	0.25	92	1.1	0.12	0.05	0.55	0.17
CoAPO-18	cubes	≤0.5	639	0.22	90	1.0	0.16	0.08	0.78	0.27
ZnAPO-18	irregular	∼1.0	96	0.01	68	1.1	0.14	0.07	0.66	0.04

a Properties determined using SEM.

b N_2_ physisorption using the BET method.

c SEM–EDS.

d Propylamine-TPD.

The elemental ratio of the synthesis gels was identical (M/(Al + P) = 0.05), and the measured elemental compositions from SEM–EDS of MAPO-18s were close to the expected values (Table 1). Propylamine TPD results, which selectively quantifies BAS, showed that only a fraction (<1/2) of the heteroatoms led to BAS formation (Table 1). Although the reason for the discrepancy remains indistinct, the influence of heteroatom and BAS density was investigated for a series of SAPO-18 and MgAPO-18 samples with M/(Al + P) ratios in the 0.03–0.06 range (Table S2, Section S2) and found to have minor influence on catalytic properties (vide infra). Hence, in the following, emphasis will be set on heteroatom characteristics and not on the density of BAS. Inspection of the desorption temperature of propylamine cracking products (propene and NH_3_, Figure S5) suggested that the acid strength of the highly crystalline samples increased in the order: SAPO-18 < CoAlPO-18 < MgAPO-18.

**Figure 2:**
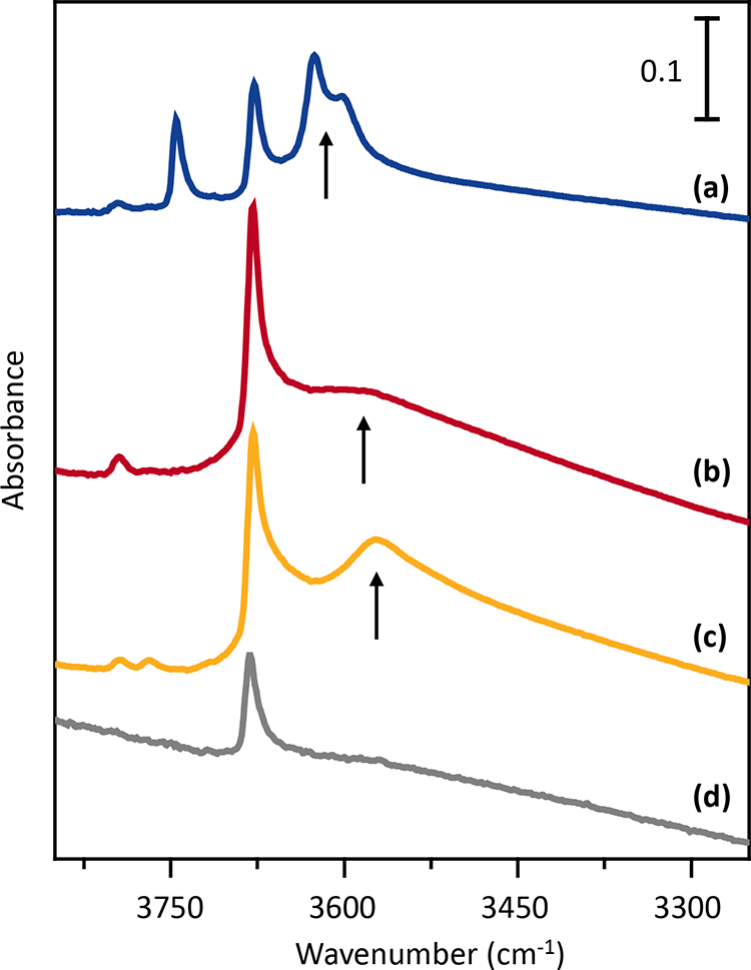
OH stretching region of IR spectra of the activated MAPO-18s. (a) SAPO-18, (b) MgAPO-18, (c) CoAPO-18, and (d) ZnAPO-18. The spectra are normalized to the pellets’thicknesses. The vertical arrows underline the downward shift of the BAS ν (OH) vibrational band.

FTIR spectroscopy was used to provide further insights into the acidity of the MAPO-18 catalysts.^38,45^ IR spectra illustrating the ν(OH) frequency region of the activated powders (treated in high vacuum at 400 °C) are reported in Figure 2. All catalysts showed a band at 3680 cm^–1^ assigned to the stretching modes of P–(OH) terminal groups. Their abundance correlated well with the small crystal size observed by SEM (Figure 1). Except for ZnAPO-18, the vibrational bands of Al–OH terminal groups were visible at 3795 and 3770 cm^–1^. An additional band at 3745 cm^–1^, due to terminal Si–OH, was observed for SAPO-18 (Figure 2 line a). Moreover, SAPO-18 showed two bands at 3627 and 3600 cm^–1^ attributed to ν Si–(OH)–Al, corresponding to BAS located in two different crystallographic positions.^38^ Coming to the MgAPO-18 (Figure 2 line b) and CoAPO-18 (Figure 2 line c) samples, IR spectra are characterized by quite broad absorption at much lower frequency, centered at 3582 and at 3570 cm^–1^, respectively.^50^ The progressive downward shifts of these bands in the series SAPO-18, MgAPO-18, and CoAPO-18, can be explained in terms of an increased BAS acidity strength in relation with the heteroatom, that follows the order Si ≪ Mg < Co.

Finally, in the case of ZnAPO-18 (Figure 2 line d), the lack of strong Brønsted sites can be explained with the formation of dense phases (Figure S3). Further insights on the acidity in the series were obtained by IR measurements coupled with CO adsorption at 77 K. Upon CO interaction, the IR spectra evolved in the ν (OH) stretching region showing BAS band downward shifts as evidenced in Figure S6. More details are reported in Supporting Information, Section S2.

### Catalytic Performance at 1 bar and 350 °C MTO Reaction Conditions

**Figure 3:**
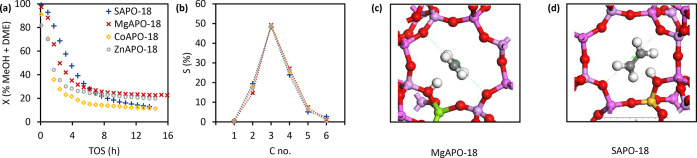
Catalytic performance of MAPO-18s at MTO reaction conditions. Reaction conditions: 350 °C, 1 bar, 0.13 bar MeOH (WHSV = 4 g_MeOH_ g_cat_^–1^ h^–1^), and 0.87 bar He. (a) Activity in terms of % sum of MeOH and dimethyl ether (DME) conversion over TOS and (b) hydrocarbon product distribution in terms of *C* no. compared at 35–40% conversion. Paraffin selectivity in all cases was less than 1%. From the reproducibility tests, the data sets were reproducible at more than 95% confidence interval and the error bars are included in Figure S15. Structure optimized 8-ring window of (c) MgAPO-18 (MgAl_23_P_24_O_96_H) and (d) SAPO-18 (SiAl_24_P_23_O_96_H) with adsorbed ethene. Ethene is perfectly centered through the 8-ring of MgAPO-18, but pushed out of the 8-ring of SAPO-18.

The influence of acid strength created by various heteroatom substitution was first evaluated under typical MTO conditions at 350 °C, 1 bar and 0.13 bar MeOH in N_2_. These conditions were selected to illustrate the “true” deactivation profile of these catalysts, meaning 100% conversion was not sustained over time-on-stream (TOS). From Figure 3a, SAPO-18 deactivated the slowest in the first 5 h of TOS, followed by MgAPO-18, ZnAPO-18, and CoAPO-18. The slower deactivation of SAPO-18 may be due to its higher number of BAS. Moreover, the possible redox activity of CoAPO-18 and ZnAPO-18 may lead to more rapid coking and faster deactivation, as in the case of CoAPO-5 and ZnAPO-5 described earlier.^36^ The superior lifetime of SAPO-18 over other MAPO-18 catalysts was also reported by other groups.^17,51^ After 5 h of TOS, the activity of the MAPO-18s appeared to stabilize at 10–25% conversion. The methanol conversion capacities of the MAPO-18s were compared with a commercial SAPO-34 (from ACS Materials) as shown in Figure S13 and the selectivity *versus* conversion plots are included in Figure S14.

Referring to Figure 3b, all MAPO-18s were selective toward propene, attaining 49% propene selectivity. On the other hand, ethene selectivity varied depending on the heteroatom and was 5% higher for SAPO-18 than for MgAPO-18. Correspondingly, butenes and pentenes selectivities were lower for SAPO-18. This difference could be assigned to a change in the steady-state HC pool composition, yielding more arenes and ethene in SAPO-18 *versus* the more strongly acidic M(II)APO-18 catalysts (Figure 2). Such an effect was previously observed for the AFI topology (a 1D, 12-ring straight channel topology with less diffusion restrictions).^36^ It was concluded that the stronger Brønsted acids [M(II)APO-5s] produce more propene and butenes, typical alkene cycle products, whereas M(IV)APO-5 (*i.e.*, SAPO-5) produces more ethene, aromatics, and alkanes, typical arene cycle products.^36^ This conclusion was recently supported by propene and benzene methylation experiments performed over Mg-, Si-, and Zr-APO-5. They revealed a higher selectivity toward propene methylation (relative to benzene methylation) over the more strongly acidic MgAPO-5 material.^52^ Finally, in the case of the AEI materials studied here, we cannot exclude the possibility that the slightly distorted 8-ring windows in M(II)APO-18 compared to Si(IV)APO-18 impacts product selectivity, by facilitating C_3+_^=^ product diffusion (Figure 3c,d).^53,54^

### Reactive Feeds at 20 bar and 350 °C

**Figure 4:**
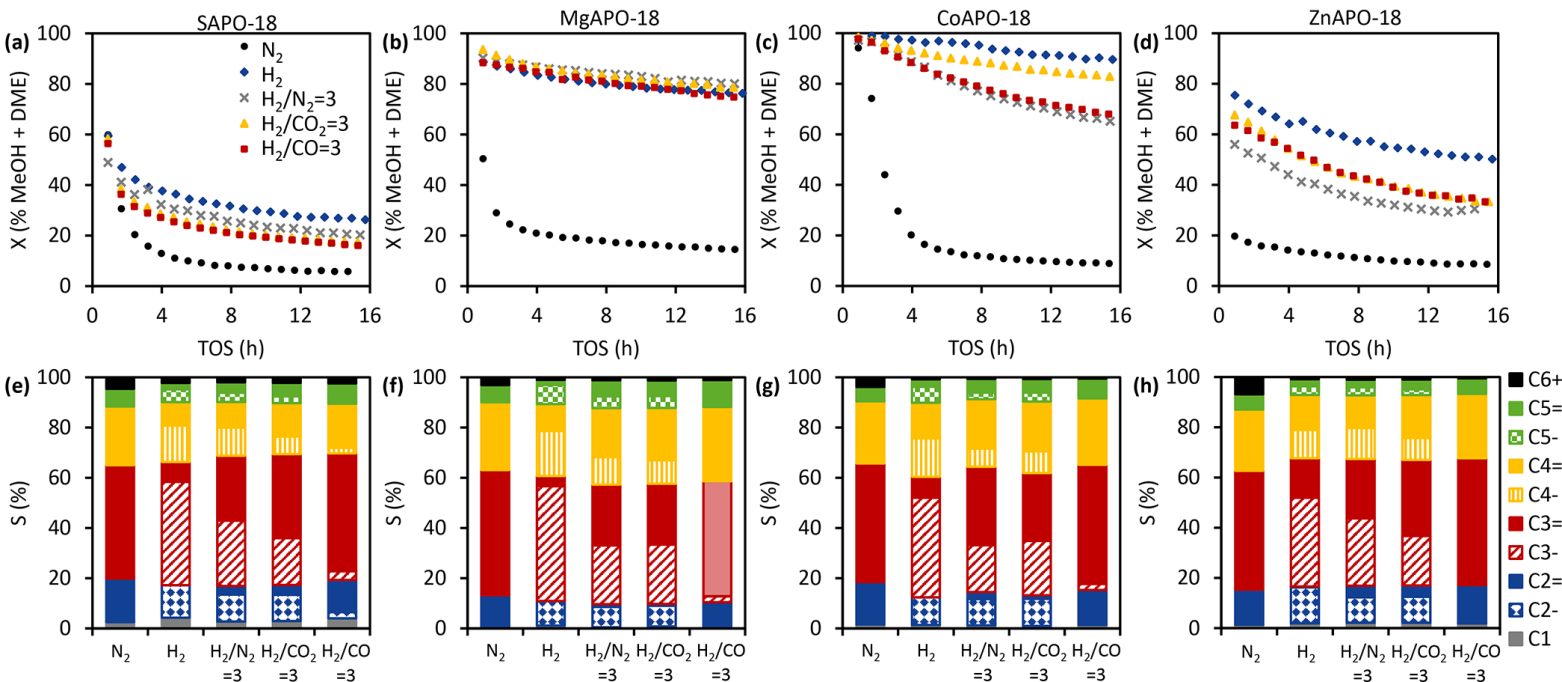
Catalytic performance of MAPO-18s at 350 °C, 20 bar, 1 bar MeOH (WHSV = 2.5 g_MeOH_ g_cat_^–1^ h^–1^), 0.6 bar Ar internal standard, 18.4 bar N_2_ or H_2_, or H_2_/X = 3 (in which X = N_2_, CO_2_, or CO), and GHSV ≈ 16 000 mL_total flow_ mL_cat_^–1^_._ H^–1^. (a–d) Activity in terms of % sum of MeOH and DME conversion over TOS for (a) SAPO-18, (b) MgAPO-18, (c) CoAPO-18, and (d) ZnAPO-18. (e–h) Product selectivity at TOS = 10 h for (e) SAPO-18, (f) MgAPO-18, (g) CoAPO-18, and (h) ZnAPO-18. From the reproducibility tests, the data sets were reproducible at more than 95% confidence interval and the error bars are included in Figure S16.

The influence of various heteroatom substitutions was next evaluated at higher reaction pressure of 20 bar and relevant reactants N_2_, H_2_, CO, and CO_2_ were co-fed with methanol. The results are presented in Figure 4 and Table 2 and revealed important reaction atmosphere effects. Detailed catalytic performance and reproducibility checks are presented in Figures S15 and S16, respectively. The increase in pressure from 1 bar to 20 bar (0.13 *vs* 1 bar MeOH in inert N_2_ feed) had negligible influence on the deactivation profiles. All MAPO-18s deactivated strongly in the first 5 h of TOS and their activities subsequently stabilized at 10–20% conversion. The commercial SAPO-34 was also tested at 350 °C, 20 bar, MeOH in N_2_ feed, and it deactivated completely after 3 h of TOS (Figure S17). Propene selectivity over MgAPO-18 was the highest at 49%, followed by CoAPO-18 and ZnAPO-18 at 47%, and SAPO-18 at 44%. SAPO-18 showed higher ethene and C_6+_ selectivities in comparison to the M(II)APO-18s.

**Table 2:** MeOH Conversion Capacity and Olefins-to-Paraffins Ratios at 350 °C, 20 bar, 1 bar MeOH (WHSV = 2.5 g_MeOH_ g_cat_^–1^ h^–1^), 0.6 bar Ar Internal Standard, 18.4 bar N_2_ or H_2,_ or H_2_/X = 3 (in which X = N_2_, CO_2_, or CO^a^)

	(MeOH + DME) conv. capacity after 16 h (g_MeOH_ g_cat_^–1^)					olefins/paraffins (C_2–4_^=/–^) at 10 h TOS				
	N_2_	H_2_	H_2_/N_2_ = 3	H_2_/CO_2_ = 3	H_2_/CO = 3	N_2_	H_2_	H_2_/N_2_ = 3	H_2_/CO_2_ = 3	H_2_/CO = 3
SAPO-18	5.1	13.1	10.6	9.6	9.1	40.2	0.3	0.8	1.4	9.6
MgAPO-18	7.6	30.2	30.2	30.3	29.1	101	0.2	1.1	1.1	22.8
CoAPO-18	8.4	34.1	28.5	32.4	28.7	81.5	0.3	1.5	1.2	22.7
ZnAPO-18	4.6	22.0	13.7	17.0	16.4	95.8	0.5	0.8	1.3	91.2

a Please note that the conversion levels differ between the catalysts (*cf.*Figure 4).

Next, the general strategy of co-feeding H_2_ with methanol at high pressures to improve the catalyst lifetime was proven to be effective, in accordance with the earlier literature.^32−35^ This improvement in lifetime was usually obtained at the expense of lower conversion levels or steep initial deactivation, due to the higher production of paraffins as terminal products.^33,34^ Remarkably, beyond prior studies, a comparison between the four materials showed that less H_2_ is needed to acquire high, semistable activity over MgAPO-18 than over the other three materials (Figure 4a–d). All three M(II)APO-18 catalysts showed higher conversion levels in H_2_ than those in N_2_ environment, despite the increased production of paraffins. Furthermore, the M(IV) heteroatom substituted SAPO-18 was the only catalyst which showed a steep initial deactivation. This steep initial deactivation was reported recently by Shi *et al.* for both SAPO-34 and SSZ-13 under high pressure H_2_ co-feeding, although the activity of the more acidic SSZ-13 later stabilized at ∼15% conversion.^34^ This result may be linked to the parameters discussed above, such as the potential preference of M(II)APO materials for the alkene cycle mechanism,^52^ or facilitated product diffusion with distorted windows (Figure 3c,d). In both cases, less hydrogen addition may be needed to balance the steady-state HC pool composition in a favorable direction for the non-transition metal catalyst with stronger acid sites, MgAPO-18, followed by Co- and Zn-APO-18. The increase in turnover numbers upon hydrogen addition to the MTO feed, due to the hydrogenation of the coke precursors formaldehyde and dienes, were previously concluded experimentally and theoretically.^32−34,55^ Their hydrogenation rates is strongly favored over alkene (and particularly arene) hydrogenation, and all hydrogenation rates are favored by a higher BAS strength.

H_2_ does not only hydrogenate coke precursors, but it also hydrogenates the olefinic products hence decreasing the olefins-to-paraffins ratio (Table 2).^33,35,56^ Applicable to all MAPO-18 catalysts, the olefins-to-paraffins ratio was lowest for C_2_, followed by C_3_ and then C_4_ (Figure 4e–h, Table S3). This is in contrast to the mechanistic studies performed on zeolites with CHA, AEI, FER, and BEA topology.^33^ Arora *et al.* observed the lowest hydrogenation rate for ethene in all zeolites and higher formation of propane especially for SSZ-39, which is the zeolite version of MAPO-18s.^33^ For the isostructural CHA catalysts, propane selectivity was notably higher than ethane and butanes, and SSZ-13 with stronger acidic strength resulted in higher hydrogenation rate constants than SAPO-34.^34^ According to DeLuca *et al.*, the reaction barrier to alkene hydrogenation decreases with an increasing chain length and stabilization of the intermediate carbocation-like species.^55^ Comparison of the SSZ-13 zeolite to the isostructural SAPO-34 material further showed a positive relation between the acid strength and hydrogenation rates, and this acid strength effect is more significant than topology (ZSM-5 *vs* SAPO-34).^55^ In the current case, the preferred hydrogenation of ethene suggests that the heteroatoms are preferably located in the 8-ring windows of the AEI topology: strong confinement effects for small-molecule conversion have recently been demonstrated for C_2_^=^–C_4_^=^ methylation in 1D 10-ring ZSM-22 (TON)^57^ and for methanol carbonylation reactions in the 8-ring pockets of MOR.^58,59^

The simultaneous hydrogenation of olefinic products due to H_2_ co-feeding calls for an improvement to this strategy for enlarging lifetimes. A possible solution suggested by the Liu group is the co-feeding of H_2_O with H_2_ and methanol.^35^ As H_2_O may lead to material degradation and was calculated to compete for BAS adsorption with methanol, potentially yielding lower conversion rates,^60^ we suggest co-feeding CO with H_2_ and methanol instead. Although this approach resulted in a negative effect on the semistable conversion level for most materials (Figure 4a–d), it also led to a dramatic, positive effect on the olefins-to-paraffins ratios for all MAPO-18 catalysts (Figure 4e–h). Strikingly, MgAlPO-18, the non-transition metal material with the highest acid strength, showed similar, enhanced conversion level as with H_2_ co-feed, with olefins-to-paraffins ratios higher than 22 for the C_2_–C_4_ products (Table 2). This result points to a distinct role of CO in the MTO reaction, as reflected in the ongoing debate on whether CO is a co-catalyst or a stoichiometric reactant in the initiation of the dual-cycle MTO mechanism,^39−44^ and in recent CH_3_OH and CO co-feed studies that demonstrated *C* insertion from CO in HC products over ZSM-5.^61^ Complementary tests in which CO was co-fed with methanol over the Si-, Mg-, and Co-APO-18 catalysts, without H_2_ co-feed, showed negligible impact on the activity and product distribution (Figures S18–S20). These results suggest either that CO does not take part in the reaction under the conditions studied here or that the cavity-window structure of the AEI topology leads to a diffusivity-dominated steady-state HC pool composition that masks its contribution in the absence of H_2_ co-feed.

Identical experiments of co-feeding CO_2_ with H_2_ and methanol were performed over the MAPO-18s as well. Although there were more olefins produced with CO_2_/H_2_ co-feed than pure H_2_ co-feed (Figure 4e–h), this was likely due to the lower H_2_ partial pressure. This hypothesis was confirmed with reference experiments carried out in N_2_/H_2_ co-feed environment in which the H_2_ partial pressure was kept constant, as similar olefins-to-paraffins ratios were attained (Table 2, Figure S15). These results underline the unique role of CO in the MTO process, which will be covered in detail in the following section.

### Role of CO in the MTO Process

The limited deactivation coupled with high selectivity toward olefins suggests that CO suppressed the hydrogenation of the olefinic products but not the coke precursors. In the recent literature, the hydrogenation rate constants of coke precursors such as formaldehyde and butadiene were measured to be at least 2 orders of magnitude higher than that of propene and ethene in high pressure H_2_ studies, and this was proposed to be the origin of the lifetime improvement with limited increase (2–3 times) in paraffin selectivity.^33,34^ This is not the case for the reaction conditions used here because paraffin selectivity increased dramatically for all catalysts (Table 2). A wider range of reaction conditions, including different CO/CO_*x*_ feed ratios and lower methanol partial pressure, were then explored with SAPO-18 (Figure S21). At those conditions, the ratio between H_2_ and hydrocarbon products was higher and almost 100% paraffin selectivity was obtained in the presence of CO_2_ exclusively (CO/CO_*x*_ = 0 in Figure S21). A clear decrease in paraffin selectivity was observed when the CO/CO_*x*_ ratio was increased. With lower MeOH partial pressure, that is, higher H_2_/MeOH ratio, paraffin selectivity was also close to 100% in the presence of CO_2_, yet less than 20% in the presence of CO. This affirmed the inhibition of olefin hydrogenation due to the presence of CO.

**Figure 5:**
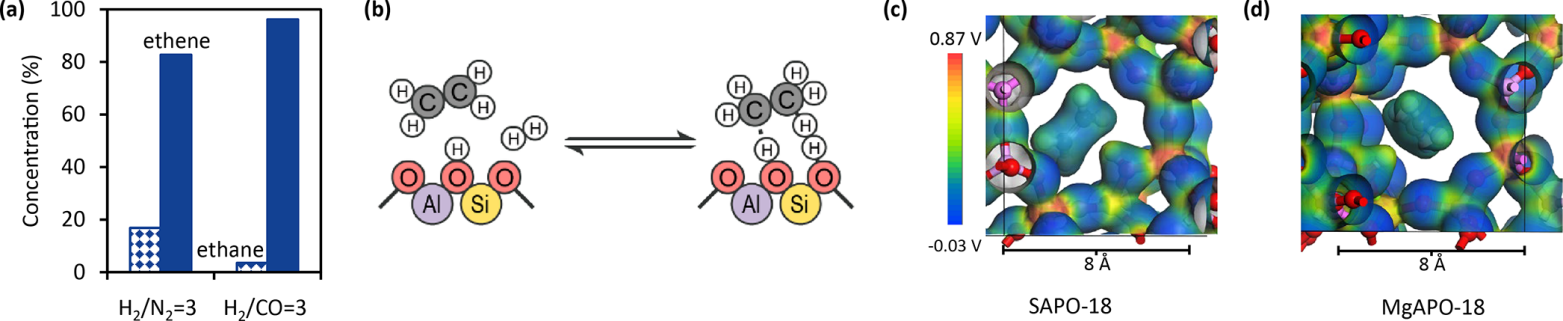
Mechanistic insights of the interaction between ethene and CO. (a) Ethene hydrogenation over SAPO-18 with N_2_ or CO co-feeding at 350 °C, 10 bar, 0.25 bar ethene, and WHSV = 1.0 h^–1^. (b) Illustration of ethene hydrogenation precursor states in SAPO-18, adapted from Hibbitts *et al.*(55) Optimized electron cloud distribution in (c) SAPO-18 and (d) MgAPO-18 with ethene, showing the more polar character of the Mg–O bond compared to the Si–O one. The atom positions are the same as in Figure 3.

To further substantiate this conclusion, ethene and H_2_ were co-fed over SAPO-18 with CO or N_2_ at 350 °C, 10 bar, and 0.25 bar ethene (WHSV = 1.0 h^–1^). From Figure 5a, the ethane concentration decreased ∼5 times upon switching from N_2_ to CO co-feeding with H_2_, and this reduction coincided with the drop in ethane selectivity from 11 to 2% during the MTO experiments (Figure 4). Thus, in addition to the discussed roles of CO in the initiation of the dual-cycle MTO mechanism or insertion into the aromatic products, these results demonstrate that CO also plays a role in inhibiting olefin hydrogenation, which is illustrated in Figure 5b. Returning to MTO conditions, catalytic stability was largely preserved when CO was added to the H_2_ co-feed (Figure S21). Hence, CO appeared not to affect the hydrogenation of diene or aromatic precursors.

Notably, CO is a weak base, which scarcely competes with methanol or DME for adsorption on BAS. Hence, initial conversion levels are maintained in the presence of the syngas co-feed, as observed in Figure 4. However, in terms of its much higher concentration (>5 times CO/alkene ratio) during co-feed experiments, CO may compete favorably with alkenes for adsorption on BAS, thereby distorting the precursor state for alkene hydrogenation reactions, leading to the observed improvements in olefins-to-paraffins ratios (Figures 4, 5). The seemingly contradictory effect of CO on alkene hydrogenation *versus* diene or aromatic hydrogenation could have multiple reasons. Van Speybroeck and co-workers performed density functional theory (DFT) calculations and derived protonation enthalpies for alkenes, cyclopentadiene, and benzene derivatives in ZSM-5 at 500 °C.^21^ They revealed that the protonation enthalpy is positive for alkenes and generally negative for cyclopentenes and benzene derivatives. Longer alkyl chains and enhanced branching generally led to higher proton affinities, hence promoting the interaction with BAS compared to light olefins and CO. Another contributing factor is the narrow window size of MAPO-18, which will lead to accumulation of the larger products in the cavities, hence increasing their local concentration, whereas light olefins will diffuse out. Support for the competitive adsorption of CO *versus* light alkenes is found in Figure S21, which demonstrates the CO concentration effect on alkene hydrogenation reactions. In line with this observation, Liu and co-workers recently reported that CO addition to the methanol feed over H-ZSM-5 constituted a carbonylation route to aromatics, whereas the hydrogen transfer path to aromatics was instead suppressed by CO.^61^

### Role of Acidity (SAPO-18 *vs* MgAPO-18) in the MTO Process with Syngas Co-feeding

Although the data presented in this study demonstrate strong performance improvements of MgAPO-18 compared to SAPO-18 under H_2_/CH_3_OH/CO co-feed conditions, the reason is not obvious. MTO product selectivity relies on a delicate balance between product diffusivity and kinetics of individual reactions in the dual-cycle scheme. Overall, alkene selectivity and methanol conversion capacity require high alkene methylation and cracking rates, compared to hydrogen transfer rates. In the syngas co-feed reaction scheme, the hydrogenation of formaldehyde and dienes, as well as olefins, further complicate the picture.

This research was started on the hypothesis that M(II) heteroatom substitution in zeotypes generates M–O bonds with higher polarity leading to stronger acidic sites than M(IV), that is Si, which would in turn amplify the positive influences of the reactive feeds at high reaction pressure. Theoretical predictions, based on dispersion-corrected DFT calculations, indicate a linear correlation between the BAS strength and alkene methylation activity for MAPO-X materials (M = Mg, Zn, Si, Ge, or Ti; X = 5, 18, or 34), with different slope for each topology.^62^ A similar correlation for benzene methylation activity as well as the promotion of propene *versus* benzene methylation rates over MgAPO-5 *versus* SAPO-5 was recently confirmed experimentally.^52^ Experimental studies further suggest that cracking reactions are promoted by high acid strength^63−65^ and so are formaldehyde, diene, and alkene hydrogenation reactions.^55^ The above results provide confidence that this is indeed the case, but absolute conclusions could not be made due to the differences in heteroatom uptake despite identical synthesis gel composition and the lack of control in specific heteroatom location. Therefore, a series of SAPO-18 and MgAPO-18 with varying heteroatom loadings were prepared to elucidate the acidity–performance relations.

**Figure 6:**
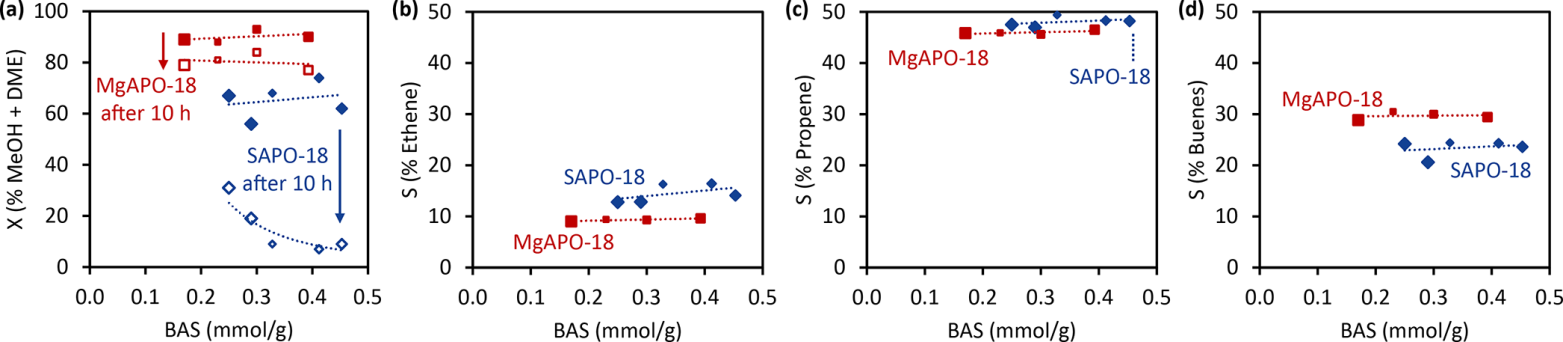
Catalytic performance of SAPO-18 and MgAPO-18 with varying BAS densities. Reaction conditions: 350 °C, 20 bar, 1 bar MeOH (WHSV = 2.5 g_MeOH_ g_cat_^–1^ h^–1^), 0.6 bar Ar internal standard, 18.4 bar H_2_/CO = 3, and GHSV ≈ 16 000 mL_total flow_ mL_cat_^–1^ h^–1^. (a) Activity in terms of % sum of MeOH and DME conversion and (b–d) C_2_–C_4_ olefin selectivity as a function of BAS density. The symbol size correlates to heteroatom loading (from *M*/*T* = 0.02 to 0.05) and lines are added to guide the eye.

From Figure 6a, the higher activity and stability of the stronger acidic MgAPO-18 over SAPO-18 catalysts were upheld over a range of BAS density and heteroatom content (see Supporting Information for extra IR characterization details, Figure S11). This indicates the stronger influence of acidic strength on performance in comparison to density of acidic sites, thus justifying our motivation to use M(II) heteroatom substitution. Product selectivities appeared to be independent of BAS density and heteroatom loading (Figure 6b–d), but with clear distinctions between SAPO-18 and MgAPO-18. Notably, MgAPO-18 enhanced the production of butenes whereas SAPO-18 produced more ethene. Furthermore, all MgAPO-18 samples maintained high activity after 10 h of TOS whereas the SAPO-18 samples deactivated strongly, hence supporting the conclusions made in earlier sections. Catalytic performance over TOS for all samples is included in Figure S22. The role of acidic strength in high pressure H_2_ co-feeding MTO process was recently studied by the Bhan group with isostructural SAPO-34 zeotype and SSZ-13 zeolite with BAS density of 0.92 and 0.44 mmol/g, respectively.^34^ There is agreement that the stronger acidic sites deactivated slower in high pressure H_2_ co-feeds, and we further established acidic strength rather than density to be a key performance descriptor.

Although the positive influence of syngas co-feeding was amplified with the M(II)APO-18s, pointing to the influence of acid strength, the reason remains elusive. As cited above, prior studies indicate that alkene and benzene methylation reactions, alkene cracking, as well as alkene, diene, and arene hydrogenation reactions are promoted by higher acid strength, albeit to a different extent. To the best of our knowledge, no predictive model has yet been presented for acid strength influence on two other relevant reaction classes, carbonylation and hydrogen transfer reactions. However, the recent literature suggests that a LAS adjacent to a BAS promotes hydrogen transfer reactions. Employing ZSM-5 catalysts with varied BAS and LAS contents, Lercher and co-workers found that the methanol conversion rate correlated with BAS concentration only whereas the rate of hydrogen transfer reactions from methanol to an alkene correlated with LAS concentration.^13^ The Olsbye group studied methanol–butene co-reactions over ZSM-5 and found that LAS alone had negligible activity for hydrogen transfer reactions, compared to BAS-containing materials.^66^ Together, these studies strongly suggest that a LAS adjacent to a BAS promotes hydrogen transfer reactions. Of particular relevance to our contribution is a recent study in which Gascon and co-workers studied the effect of adding Lewis acidic alkaline metal ions (Ca^2+^ and Mg^2+^) to ZSM-5. They found a strong, positive influence of Ca^2+^ and Mg^2+^ counterions on its alkene selectivity and conversion capacity for the MTO reaction. Extensive experimental and theoretical studies suggested that the counterion reduced the alkene and arene methylation rates over ZSM-5 by more than an order of magnitude, and moreover, arene methylation rates were more strongly reduced than alkene methylation rates, thereby leading to the observed propene selectivity and conversion capacity improvements.^21,63^ Interestingly, propene and benzene methylation studies of the same system suggested that the rate of hydrogen transfer reactions, relative to the methylation rates, is higher in the LAS-containing materials.^21^ This result is in line with the studies cited above for pristine ZSM-5.^13,66^

Returning to the MAPO-18 materials studied here, two types of Lewis acid sites were detected by FTIR spectroscopy: *M^2+^ion exchanged onto BAS.* M^2+^ counterions were observed in the CoAlPO-18 sample and in the MgAlPO-18 sample with highest Mg^2+^ content (*cf.*Figure S6), yet not in the MgAlPO-18 samples with lower Mg contents (Figure S12). Overall, the substantially lower turnover rates reported for ion-exchanged ZSM-5 compared to pristine ZSM-5 in the literature,^21^ together with the similar conversion-selectivity performance observed in this contribution for MgAlPO-18 with different Mg content (*cf.*Figure 6), may suggest that Lewis sites corresponding to M^2+^ counterions have minor influence on the observed catalyst performance in our contribution. *Extra-Framework M(II) or M(III) Cations*. Extra-framework cations were observed in the CoAlPO-18 sample (Figure S6).

An additional source of Lewis acidity, known from the prior literature, is the Lewis acid character of the M(II)–O lattice bonds (*cf.* electron distribution plots in Figure 5c,d). Such Lewis acid sites have previously been observed by Thomas and co-workers and by Catlow and co-workers, using acetonitrile as the probe molecule. The authors concluded that interaction of the MAPO-18 materials with this strongly basic probe molecules led to breaking of the M(II)–(OH)–P bond and hence to the loss of the BAS.^37,38,67,68^ Overall, LAS could influence the catalytic properties of BAS by the presence of two next-neighboring M(II) sites (−M–O–P–O–M−), where one M(II) would constitute the BAS and the other M(II) would form a (partial) extra-framework LAS site. High M(II) concentrations would increase the probability of two adjacent BAS sites being transformed to a BAS–LAS pair. In the current case, the similar performance of MgAlPO-18 catalysts with different acid site concentration (Figure 6) may suggest that even such LAS have little effect on product selectivity. However, the impact of the cavity-window structure of the AEI topology in tuning HC pool composition and effluent concentrations by restricting product diffusivities should not be underestimated (Figure 3).^6,54,69,70^

Returning finally to the effect of BAS strength, the selectivity of the more strongly acidic MgAPO-5 material toward alkene (*vs* arene) methylation,^36,52^ combined with the slightly larger window size of MgAPO-18 compared to SAPO-18 (Figure 3) are in line with and could explain the favorable performance observed for MgAPO-18 under high pressure MTO conditions with reactive co-feeds.

## Conclusions

We introduced M(II)APO-18s, in particular MgAPO-18, as favorable methanol to olefins catalysts for high pressure methanol conversion in the presence of reactive gases. All M(II)APO-18 (M = Mg, Co, or Zn) outperformed Si(IV)APO-18 at 350 °C, 20 bar, and 1 bar methanol partial pressure (WHSV = 2.5 h^–1^) with H_2_/CO or CO_2_ co-feeds. Product selectivity, specifically hydrocarbon and paraffin/olefin distributions, were regulated by zeotype composition and CO co-feeding, respectively. Furthermore, CO was proven to inhibit the hydrogenation of olefins without altering the stability of MgAPO-18. Acidity, in particular, acidic site strength and not density, was the main descriptor for the catalytic activity and stability. CO co-feeding with H_2_ and methanol is thus an effective strategy to suppress the hydrogenation of olefinic products in the MTO process.

## Supporting Information Available

Catalyst characterization (XRD, SEM–EDS, N_2_ physisorption, IR spectroscopy, and propylamine-TPD), catalyst performance, and DFT calculations (PDF)
