# Supplementary material for: MAPO-18 Catalysts for the Methanol to Olefins Process: Influence of Catalyst Acidity in a High-Pressure Syngas (CO and H2) Environment
Source: ACS Catal. 2022 Jan 11;12(2):1520–31. doi: 10.1021/acscatal.1c04694 (PMC8788383; doi:10.1021/acscatal.1c04694)
Supplement: Supplementary file 1 — cs1c04694_si_001.pdf [file cs1c04694_si_001.pdf]

Supporting Information

MAPO-18 catalysts for the Methanol to Olefins (MTO) process: Influence of catalyst acidity in high-pressure syngas (CO and H_2) environment

Jingxiu Xie[†], Daniel S. Firth[†], Tomás Cordero-Lanzac[†], Alessia Airi[‡], Chiara Negri[†], Sigurd Øien-Ødegaard[†], Karl Petter Lillerud[†], Silvia Bordiga[‡], Unni Olsbye[†,*]

[†] Centre for Materials Science and Nanotechnology, Department of Chemistry, University of Oslo, Sem Saelandsvei 26, N-0315 Oslo, Norway (E-mail: unni.olsbye@kjemi.uio.no)

[‡] Department of Chemistry, NIS and INSTM Reference Centre, Università di Torino, Via G. Quarello 15, I-10135 and Via P. Giuria 7, 10125 Torino, Italy

Contents

S1: Supporting background 2
S2: Supporting catalyst characterization 3
S3: Supporting catalyst performance 13
S4: Supporting DFT calculations 24
S5: Supporting references 35

S1: Supporting background

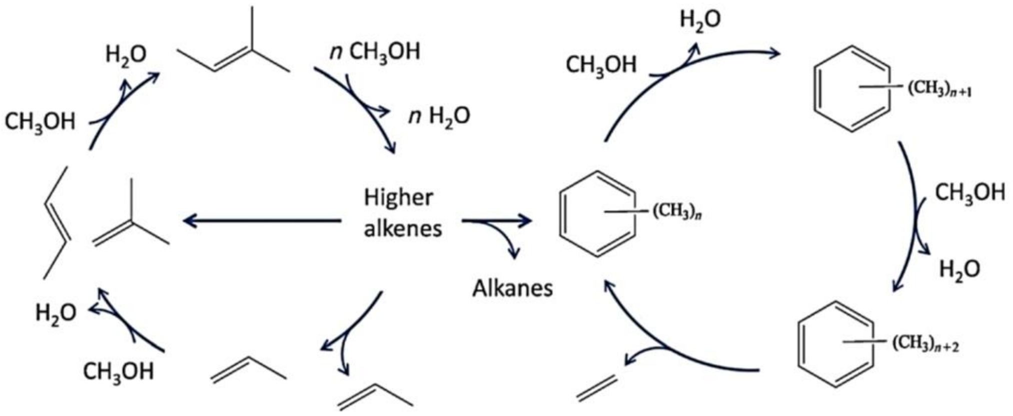

Supporting Figure 1. Dual-cycle hydrocarbon pool mechanism for the MTO reaction, from Olsbye et al.[1], with permission from John Wiley and Sons.

S2: Supporting catalyst characterization

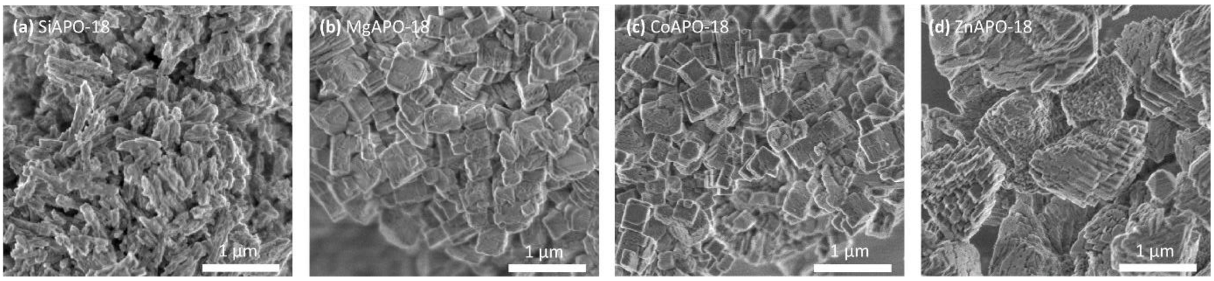

Supporting Figure 2. SEM images of calcined MAPO-18s (0.05). (a) SiAPO-18, (b) MgAPO-18, (c) CoAPO-18 and (d) ZnAPO-18.

Supporting Table 1. Atomic percent (%) obtained using SEM-EDS for the MAPO-18s. Consistent elemental composition was obtained from different locations, hence suggesting the homogeneity of the catalysts.

	Al	P	O	M
SAPO-18	12.55	12.99	67.60	1.49
	13.27	12.48	67.61	1.59
	12.42	13.13	67.84	1.61
	13.88	11.49	67.36	1.62
	12.42	12.75	68.00	1.50
MgAPO-18	12.45	14.36	67.79	1.37
	11.90	14.48	67.57	1.37
	12.22	14.02	67.31	1.35
	12.38	13.99	67.61	1.48
	12.56	13.89	67.50	1.49
	12.44	13.88	67.53	1.45
CoAPO-18	11.42	12.01	70.10	1.86
	11.59	12.08	70.11	1.85
	11.92	11.55	70.00	1.89
	11.79	11.59	70.17	1.89
	11.09	12.26	70.14	1.97
ZnAPO-18	13.00	13.94	67.18	1.82
	12.53	14.04	67.18	1.94
	12.42	14.44	67.50	1.64
	12.60	14.06	67.20	1.80

(a)

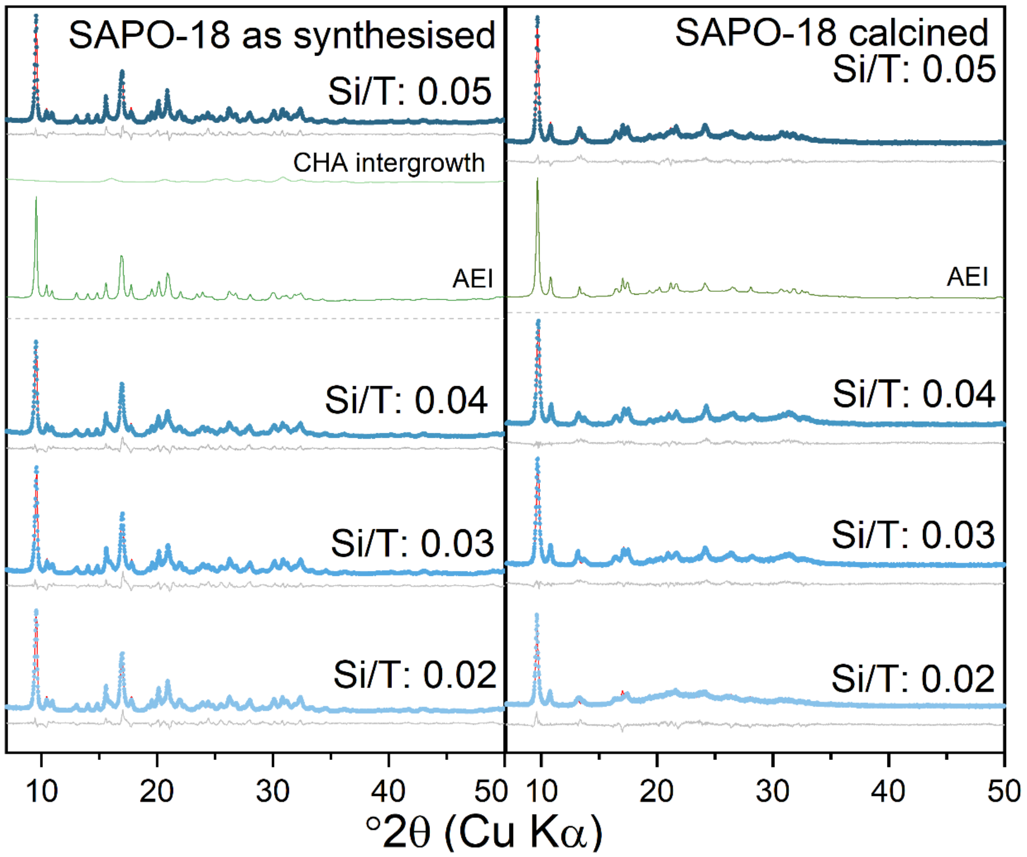
SAPO-18 as synthesised
Si/T: 0.05
CHA intergrowth
AEI
Si/T: 0.04
Si/T: 0.03
Si/T: 0.02
SAPO-18 calcined
Si/T: 0.05
AEI
Si/T: 0.04
Si/T: 0.03
Si/T: 0.02
10 20 30 40 50 10 20 30 40 50
°2θ (Cu Kα)

(b)

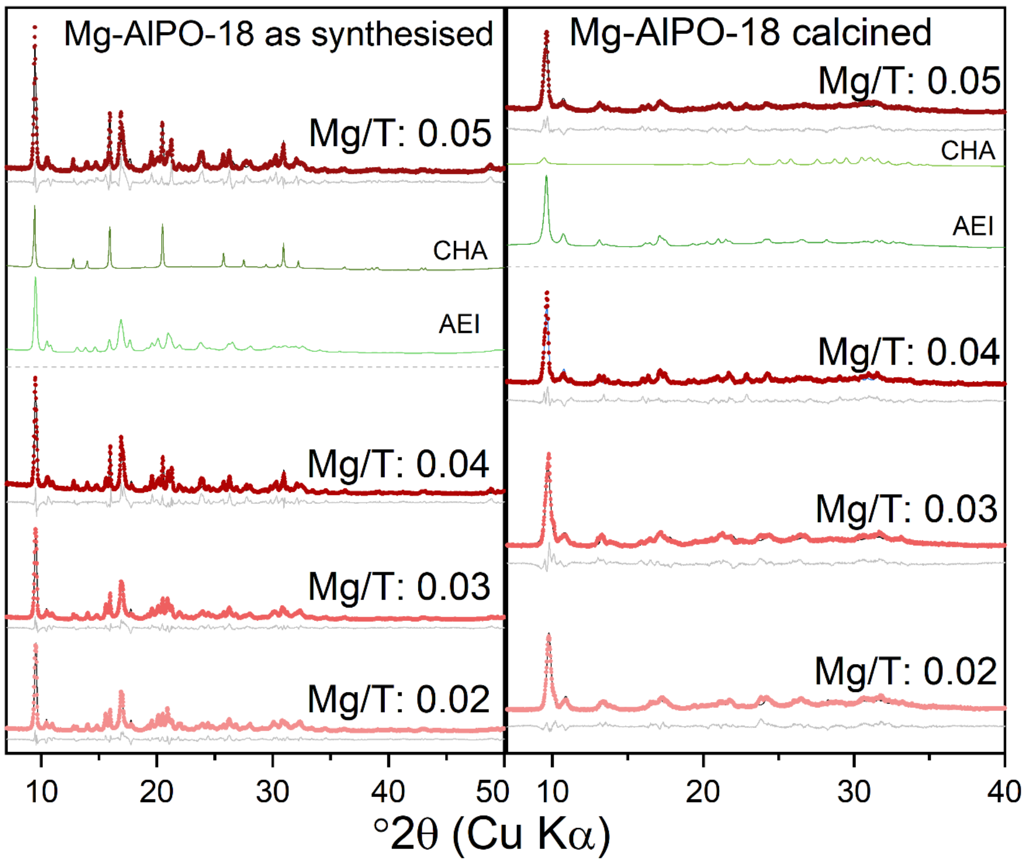
Mg-AlPO-18 as synthesised
Mg/T: 0.05
CHA
AEI
Mg/T: 0.04
Mg/T: 0.03
Mg/T: 0.02
Mg-AlPO-18 calcined
Mg/T: 0.05
CHA
AEI
Mg/T: 0.04
Mg/T: 0.03
Mg/T: 0.02
10 20 30 40 50 10 20 30 40
°2θ (Cu Kα)

(c)

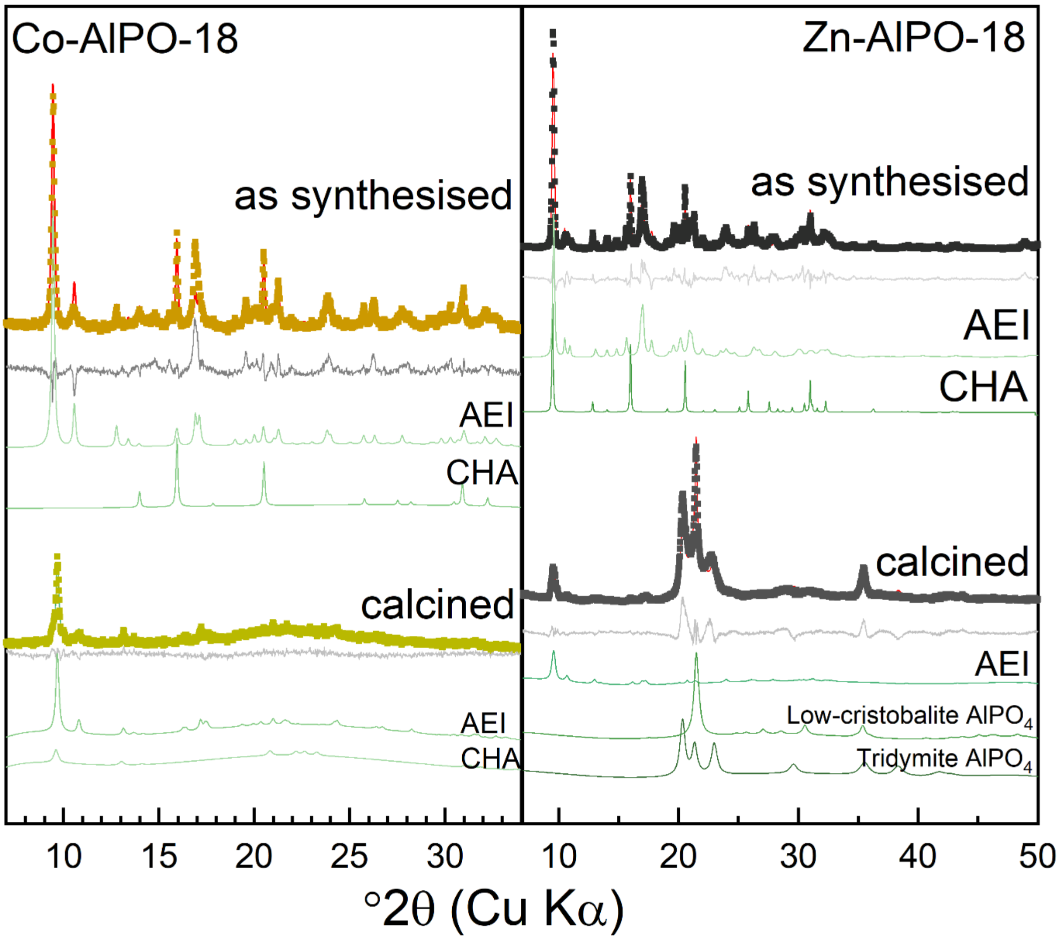

Supporting Figure 3. PXRD patterns of as-synthesized (uncalcined) and calcined MAPO-18 catalysts. (a) PXRD patterns of SAPO-18 samples as-synthesised (left panel) and calcined (right panel), at displayed Si/T atom ratios. Phase deconvolution is only reported for the Si/T: 0.05 samples, as the other samples within the series display nearly identical patterns. Measured pattern: blue dots, calculated pattern: red line, deconvoluted phases: green lines, difference (experimental - calculated pattern): grey lines., (b) PXRD patterns of Mg-AlPO-18 samples as-synthesised (left panel) and calcined (right panel), at displayed Mg/T atom ratios. Phase deconvolution is only reported for the Mg/T: 0.05 sample, as the other samples within the series contain the same phases. Measured pattern: red dots, calculated pattern: black line, deconvoluted phases: green lines, difference (experimental - calculated pattern): grey lines. (c) PXRD patterns of Co-AlPO-18 (left panel) and Zn-AlPO-18 (right panel) samples, at 0.05 M/T-atom ratios. Measured pattern: yellow (Co-AlPO-18) and grey (Zn-AlPO-18) dots, calculated pattern: black line, deconvoluted phases: green lines, difference (experimental - calculated pattern): grey lines.

The SAPO-18 samples display a pure AEI structure, with minor presence of CHA intergrowth which is regularly observed for CHA and AEI materials.[2] The Mg-AlPO-18 samples display a mixture of CHA and AEI phases as synthesized, with a minor presence of CHA still visible after calcination. The Co- and Zn-AlPO-18 samples display a mixture of CHA and AEI phases as synthesized, with a minor presence of CHA still visible in the Co-AlPO-18 sample after calcination.

The Zn-AlPO-18 decomposes upon calcination to tridymite and low-cristobalite $AlPO_4$. No separate ZnO phases are detected.

The evaluation of the samples' structure is complex due to several factors. (1) The significant phase change of the AEI materials upon calcination,[3] (2) the small domain size and high disorder in all samples (which is intentional to improve their performance as catalysts) and (3) the absence of accurate structure models. The lack of accurate structure models, and the low signal to noise ratio of the data, prevents the effective refinement of known AEI/CHA stacking faults, and the quantification of the AEI/CHA ratio in the samples where large CHA crystallites are present.

SAPO-34 is regularly obtained as cuboid crystals, whereas SAPO-18 synthesized with the DIPEA SDA tends to give elongated crystallites with diagonal facets (Figure S3). The M-AlPO-18 (M=Si, Zn) materials obtained in this study occur as disordered agglomerates of nanosized (10-200 nm) crystals with elongated shape, whereas the M-AlPO-18 (M=Mg,Co) materials occur as cuboid or prism-like particles (0.1 – 1 micron) with uneven surfaces.

Analysis of the PXRD patterns of SAPO-18 by Rietveld refinement against published structures shows a close agreement to the calculated pattern and high phase purity. For the non-calcined sample, peak broadening of certain peaks point to the presence of stacking faults.

Inspection of the PXRD patterns of Co/Mg-AlPO-18 before and after calcination reveals that the material consists of two distinct phases, AEI and CHA. The peak profiles of non-overlapping peaks from the two phases are significantly different, where the CHA peaks are sharper indicating larger diffracting domains. This was confirmed qualitatively by a combined Rietveld/Pawley fit, giving a domain size ratio of CHA/AEI of ≈2:1. Thus, the presented evidence points towards large cuboid CHA crystals, with AEI surface growth, closely resembling previously reported SAPO-18 surface crystallites on cuboid SAPO-34 crystals.[4]

In both the Mg-AlPO-18 and Co-AlPO-18 samples, the CHA phase is no longer clearly observed in the PXRD patterns after calcination, indicating a lower thermal stability of this CHA phase than pure AlPO-18. The experiments have been reproduced several times, with varying degree of CHA content before calcination, with no discernable effect on the catalytic performance. Thus, we expect that the active phase in the materials reported herein is AEI in all cases.

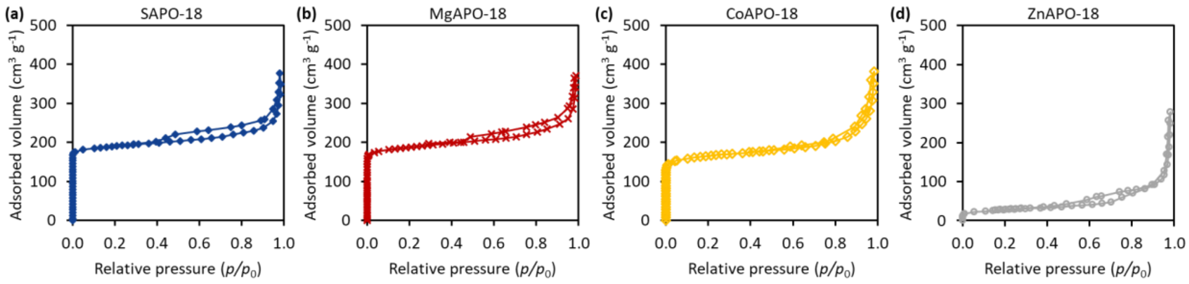

Supporting Figure 4. N_2 physisorption analysis of calcined MAPO-18s (0.05). (a) SAPO-18, (b) MgAPO-18, (c) CoAPO-18 and (d) ZnAPO-18. All MAPO-18 (except ZnAPO-18) showed a similar Type I isotherm. The ZnAPO-18 isotherm also confirmed the presence of a largely dense phase in the calcined material.

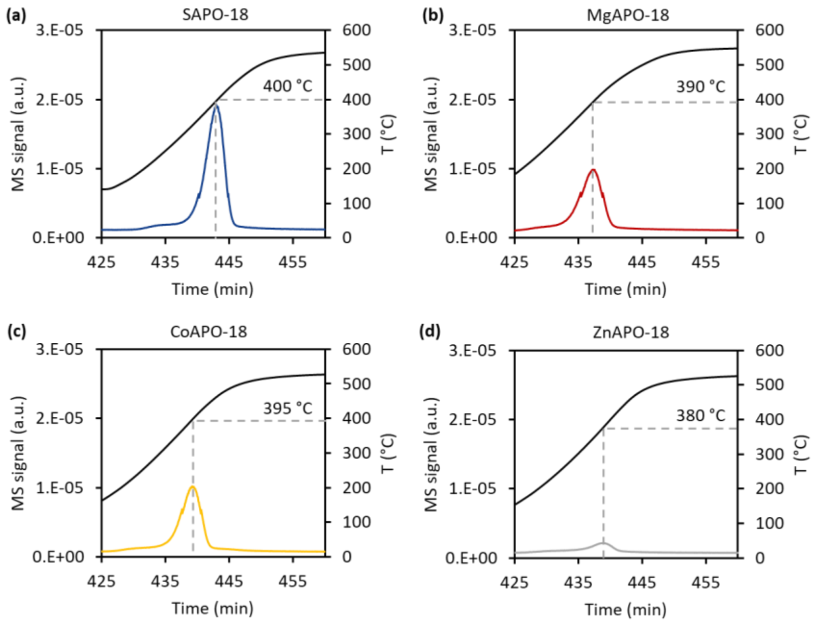

Supporting Figure 5. Propylamine-TPD analysis of calcined MAPO-18s (0.05). (a) SAPO-18, (b) MgAPO-18, (c) CoAPO-18 and (d) ZnAPO-18. Product desorption temperature is expected to be inversely proportional to the acidic strength of BAS, since its main contributor is propyl amine cracking temperature, but product diffusion rates may also influence the observed desorption temperature.

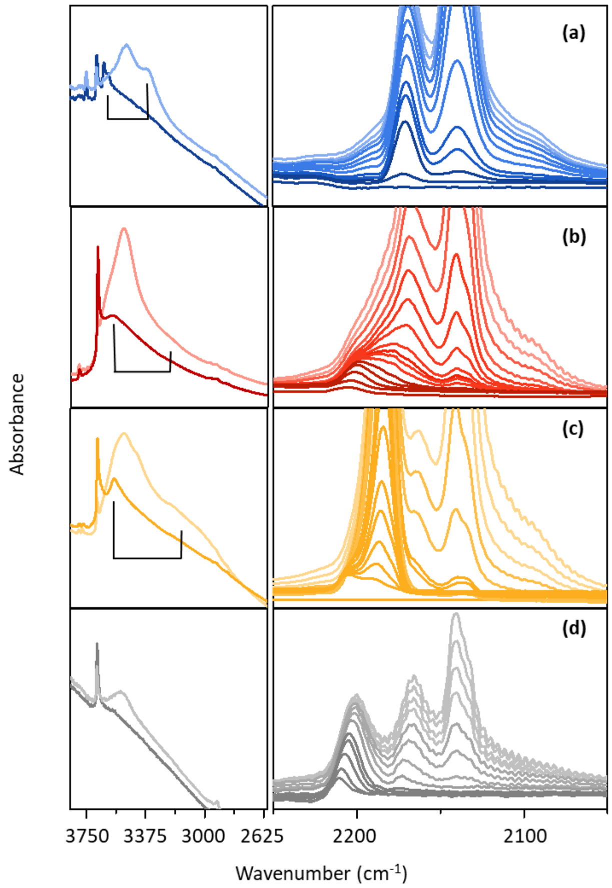

Supporting Figure 6. IR spectra of MAPOs-18: (a) SAPO-18, (b) MgAPO-18, (c) CoAPO-18, (d) ZnAPO-18 in presence of adsorbed CO at 77K at decreasing coverages. Left panels: OH stretching region (from 3800 to 2600 cm^{-1}) of activated samples (dark lines) and in presence of the highest CO coverage (light lines). Right panels: CO stretching region (from 2250 to 2050 cm^{-1}) of spectra recorded in presence of decreasing CO equilibrium pressure (from lighter to darker lines).

Left panel illustrates the effect of CO interaction towards the variety of OH groups. Upon interaction with CO in all the samples, most of the P-OH are eroded, with the parallel growth of a band at 3510 cm^{-1}, confirming the low acidic character of these species. More complex is the evolution of BAS. In particular, BAS signals are downward shifted to 3350 cm^{-1} for SAPO-18, and to 3280 cm^{-1} for MgAPO-18 respectively, following the trend observed for the ν(OH) (see main text). In case of CoAPO-18 the BAS species interacting with CO generate a very broad band extending from 3300 to 3000 cm^{-1}.

Right panels show the corresponding CO stretching range. All the samples are characterized by a strong component due to physiosorbed CO at 2140 cm^{-1} and a contribution around 2160 cm^{-1}, associated to CO interacting with the low acidic P-OH. Moreover, the interaction of CO with BAS is observed at 2170 cm^{-1} and at 2178 cm^{-1} in case SAPO and MgAPO-18 respectively. In MgAPO-18, a minor contribution is also observed at 2205 cm^{-1}, corresponding to the interaction of CO with extraframework Mg^{2+}cation. As regards the CoAPO-18, a very strong band is centred at 2180 cm^{-1}. The literature assigns this band to a convolution of different contributions,[5,6] nominally the interaction of CO with Co^{2+} Lewis acid centres with different coordination within the framework (tetracoordinated Co^{2+} at 2180 cm^{-1} and defective tricoordinated Co^{2+} at 2185 cm^{-1}) and a component assigned to tetracoordinated Co^{3+} at 2178 cm^{-1}, due to the oxidation process caused by

exposure of the sample to atmospheric moisture. From our experimental data (the evolution of the IR spectra in the OH region) we suggest that the component at 2180 cm^{-1} contains also the contribution of CO interacting with BAS, as this signal decreases together with the band extending from 3300 to 3000 cm^{-1}, while the BAS band is being restored. Finally, at low pressures of CO a small band is visible at 2210 cm^{-1}, generated by the interaction of the probe with a minor fraction of extraframework Co^{2+} cations. ZnAPO shows, apart the signal of CO liquid like and CO interacting with P-OH, a component at very high frequency (2210 cm^{-1}) suggesting the presence of highly unsaturated Zn^{2+} sites.

Supporting Table 2. Textural and acidic properties of the SAPO-18 and MgAPO-18 catalysts with varied M/T atomic ratios. SAPO-18 and MgAPO-18 were catalysts discussed in the manuscript as part of the series of MAPO-18 (M = Si, Mg, Co and Zn). SAPO-18_a to d and MgAPO-18_a to c were prepared by varying M/T ratios in the synthesis gel recipes.

	Crystal size (μm) [a]	S_{BET} (m^2/g) [b]	Elemental Composition [c]			Density of M (mmol/g_{cat}) [c]	Brønsted Acidity (mmol/g_{cat}) [d]
			P/Al	M/Al	M/T Atoms		
SAPO-18	~ 0.5	749	1.0	0.12	0.06	0.61	0.29
SAPO-18_a	~ 0.5	729	1.0	0.06	0.03	0.51	0.33
SAPO-18_b	~ 0.5	772	1.0	0.09	0.04	0.71	0.41
SAPO-18_c	~ 0.5	685	0.9	0.12	0.06	0.94	0.45
SAPO-18_d	~ 0.5	749	0.9	0.12	0.06	1.01	0.25
MgAPO-18	~ 0.5	730	1.1	0.12	0.05	0.55	0.17
MgAPO-18_a	~ 0.5	748	1.0	0.06	0.03	0.49	0.23
MgAPO-18 _b	~ 0.5	755	1.0	0.08	0.04	0.65	0.30
MgAPO-18_c	~ 0.5	741	1.0	0.11	0.05	0.81	0.39

Properties determined using [a] SEM, [b] N_2-physisorption using BET method, [c] SEM-EDS, [d] propylamine-TPD.

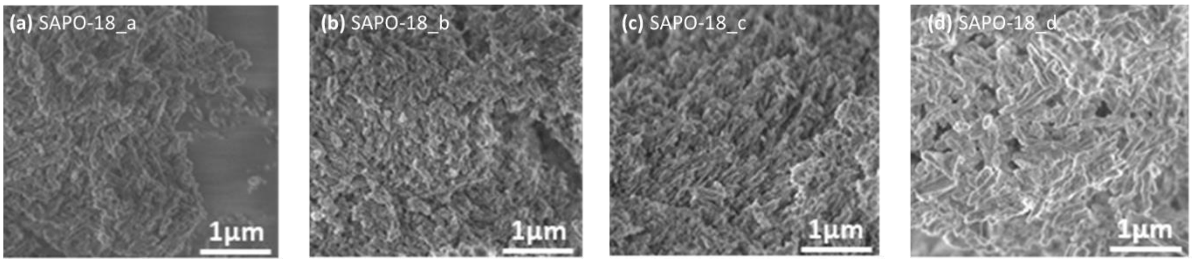

Supporting Figure 7. SEM images of calcined SAPO-18s. (a) SAPO-18_a, (b) SAPO-18_b, (c) SAPO-18_c, and (d) SAPO-18_d all showed characteristic small rods.

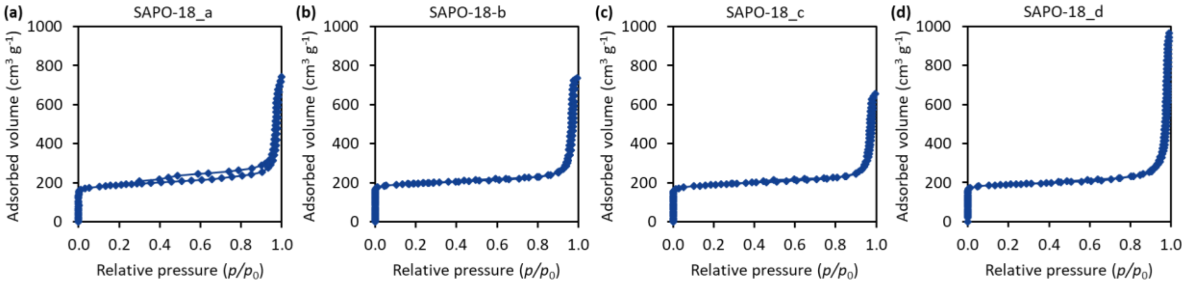

Supporting Figure 8. N_2 adsorption desorption isotherms of calcined SAPO-18s. (a) SAPO-18_a, (b) SAPO-18_b, (c) SAPO-18_c and (d) SAPO-18_d. All showed a similar Type I isotherm behavior.

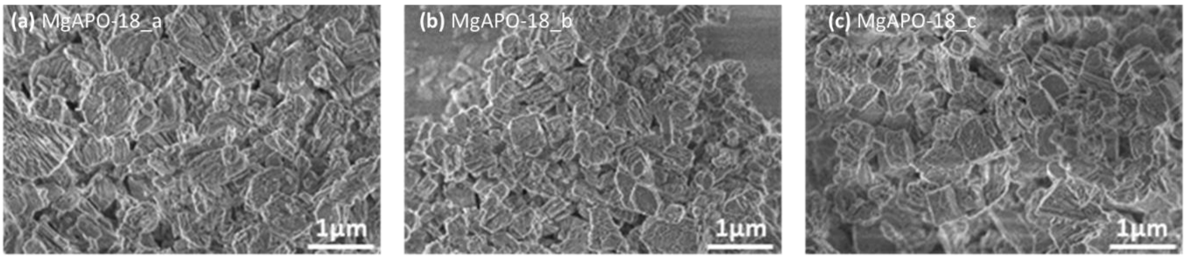

Supporting Figure 9. SEM images of calcined MgAPO-18s. (a) MgAPO-18_a, (b) MgAPO-18_b and (c) MgAPO-18_c all appeared cubic with irregular and rough surfaces. This was different to the SiAPO-18 materials and may be as a consequence of the use of magnesium in the synthesis. This suggests that the crystallization process consisted of agglomerates of smaller crystals. The size and morphology of the crystallites was consistent between samples of varying Mg-content.

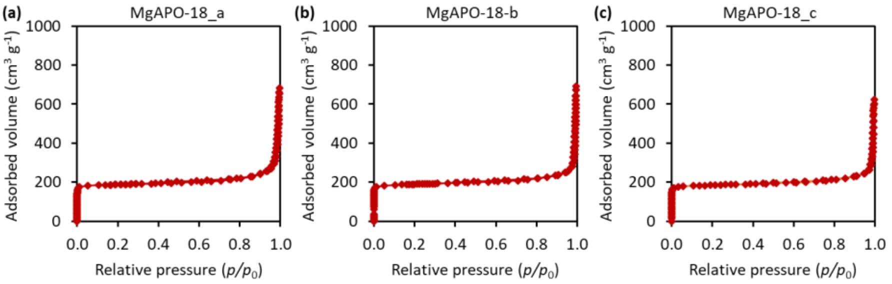

Supporting Figure 10. N_2 adsorption desorption isotherms of calcined MgAPO-18s. (a) MgAPO-18_a, (b) MgAPO-18_b and (c) MgAPO-18_c. All showed a similar Type I isotherm behavior with capillary condensation seen at high relative pressure.

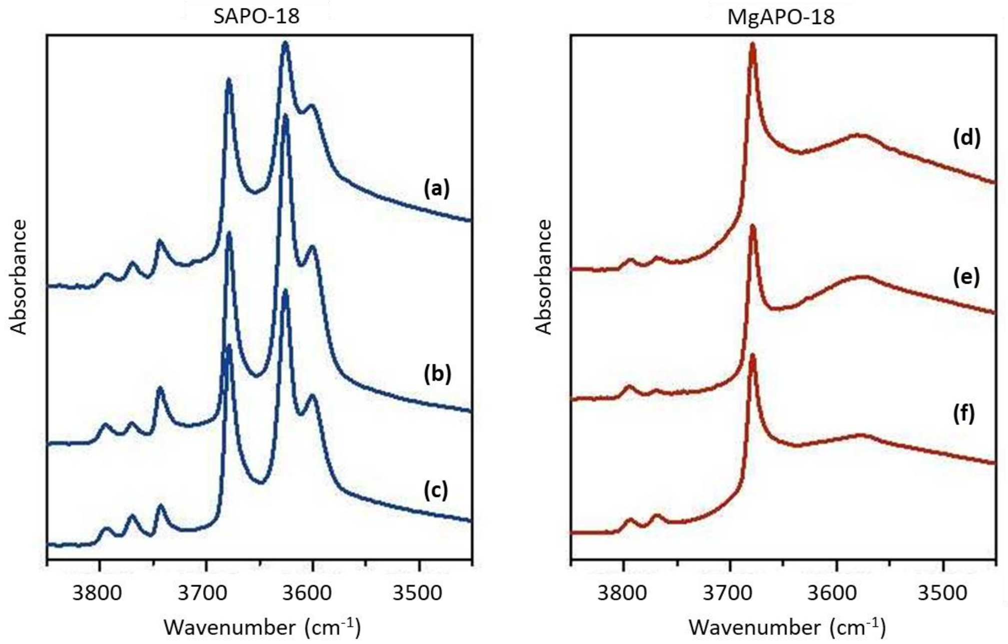

Supporting Figure 11. OH stretching region of IR spectra of activated SAPO-18s and MgAPOs. (a) SAPO-18_a, (b) SAPO-18_b, (c) SAPO-18_c, (d) MgAPO-18_a, (e) MgAPO-18_b and (f) MgAPO-18_c. The spectra are normalized to the pellets' thickness.

The IR spectra of all samples are characterized by the components already described in the main text. Notably, the variation of BAS bands intensities, moving from the bottom to the top of the series is not proportional to the increment of the heteroatom contents but it follows the trend evaluated by propylamine-TPD (Table S2). The decreased amount of BAS observed for the sample with the higher amount of Si, indicates that in this sample Si is present aggregated in islands as widely reported in the literature.[7,8] In case of MgAPO-18_a (Figure S12 below); MgAPO-18_b and MgAPO-18_c samples, CO adsorption at low temperature did not evidence the presence of any Mg^{2+} counterion: lack of the band at 2205 cm^{-1}.

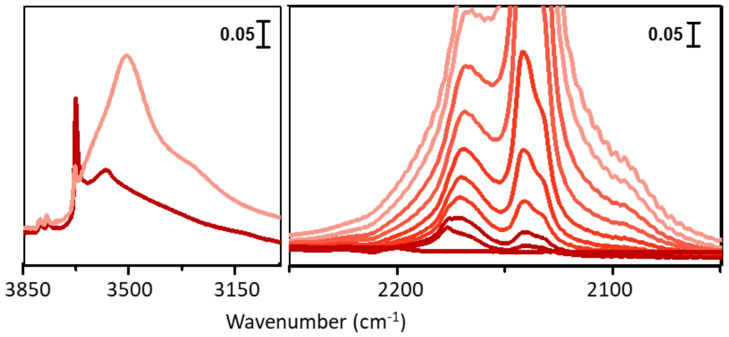

Supporting Figure 12. IR spectra of MgAPO-18_a in presence of adsorbed CO at 77K at decreasing coverages. Left panels: OH stretching region (from 3800 to 3000 cm^{-1}) of activated samples (dark lines) and in presence of the highest CO coverage (light lines). Right panels: CO stretching region (from 2250 to 2050 cm^{-1}) of spectra recorded in presence of decreasing CO equilibrium pressure (from lighter to darker lines).

S3: Supporting catalyst performance

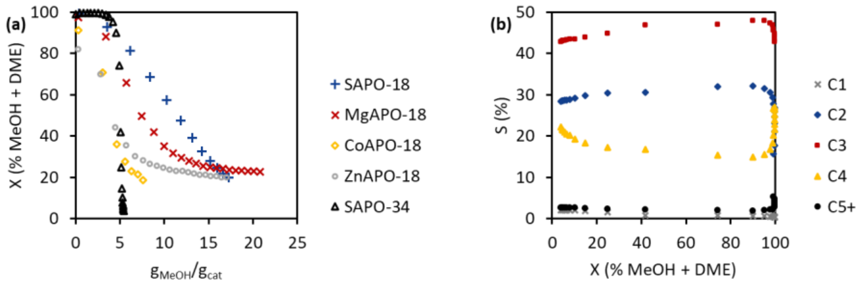

Supporting Figure 13. Catalytic performance of commercial SAPO-34 (from ACS Materials) at MTO reaction conditions. Reaction conditions: 350 °C, 1 bar, 0.13 bar MeOH (WHSV = 0.25 $g_{MeOH}\ g_{cat}^{-1}\ h^{-1}$), 0.87 bar He. (a) Activity in terms % sum of MeOH and DME conversion over MeOH conversion capacity. The MAPO-18s were tested at identical conditions except with a higher WHSV (MeOH) = 4 $g_{MeOH}\ g_{cat}^{-1}\ h^{-1}$. (b) Selectivity vs. conversion of SAPO-34. Conversion variation was due to catalyst deactivation during runtime.

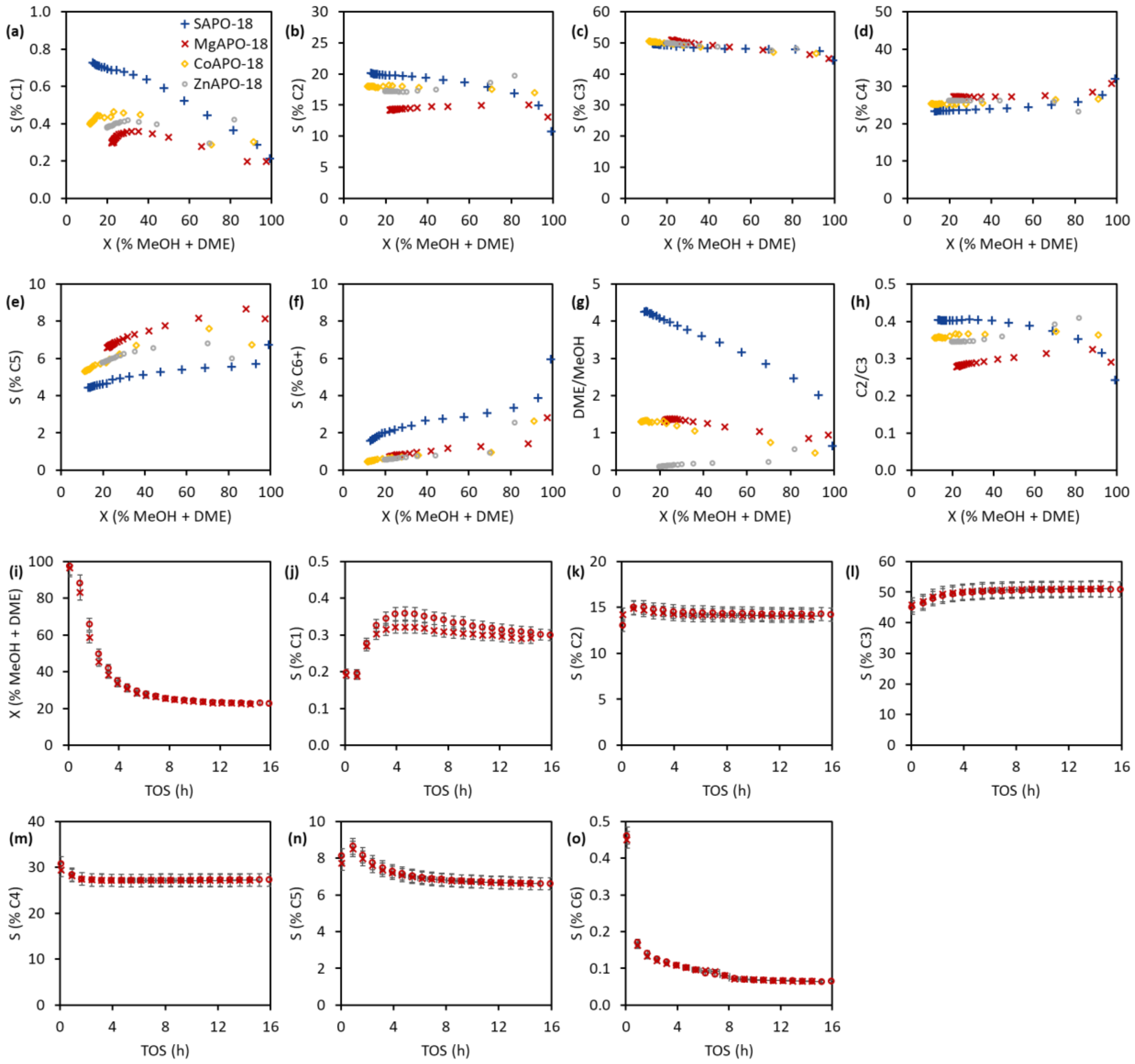

Supporting Figure 14. Selectivity vs. conversion of MAPO-18s at MTO reaction conditions (a to h) and reproducibility runs of MgAPO-18 (i to o) with error bars at 95 % confidence interval. Reaction conditions: 350 °C, 1 bar, 0.13 bar MeOH (WHSV = 4 g_{MeOH} g_{cat}^{-1} h^{-1}), 0.87 bar He. Conversion variation was due to catalyst deactivation during runtime.

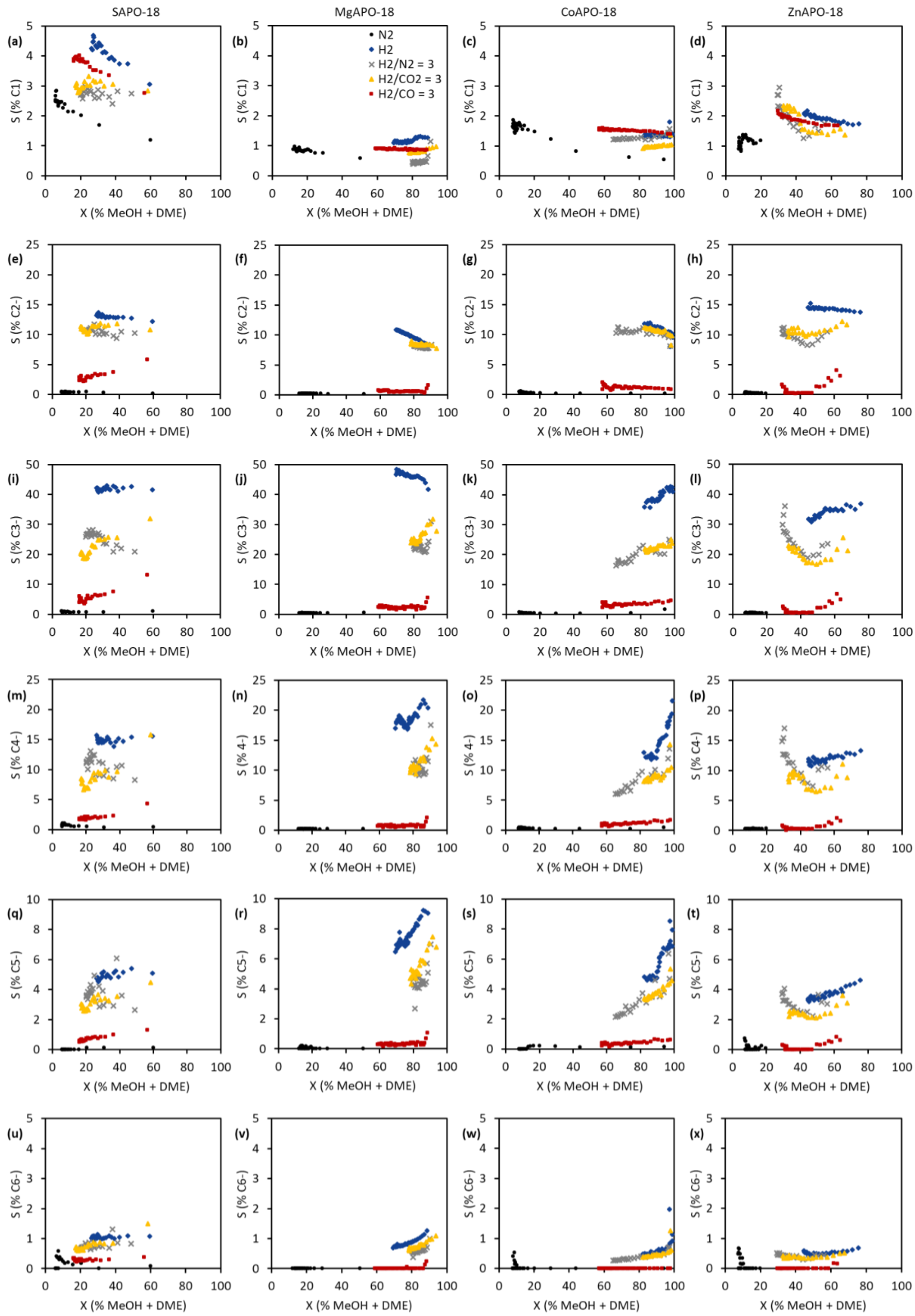

Supporting Figure 15. Paraffin selectivity vs. conversion of MAPO-18s in various reaction feeds. Reaction conditions: 350 °C, 20 bar, 1 bar MeOH (WHSV = 2.5 g_{MeOH} g_{cat}^{-1} h^{-1}), 0.6 bar Ar internal standard, 18.4 bar N_2 or H_2 or $H_2/X = 3$ (in which X = N_2, CO_2 or CO), GHSV ≈ 16 000 $mL_{total\ flow}$ mL_{cat}^{-1} h^{-1}. Conversion variation was due to catalyst deactivation during runtime.

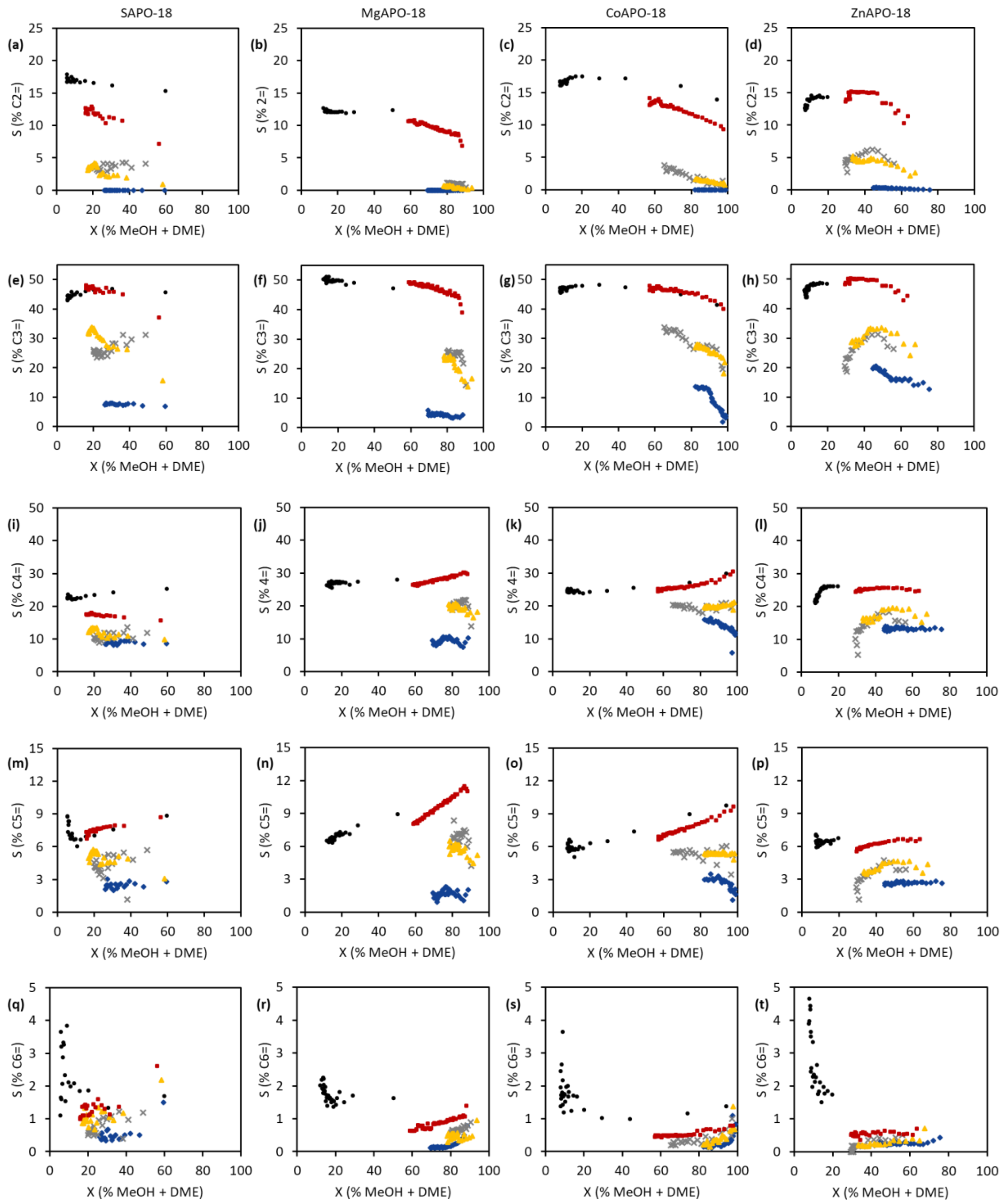

Supporting Figure 15 (II). Olefin selectivity vs. conversion of MAPO-18s in various reaction feeds. Reaction conditions: 350 °C, 20 bar, 1 bar MeOH (WHSV = 2.5 g_{MeOH} g_{cat}^{-1} h^{-1}), 0.6 bar Ar internal standard, 18.4 bar N_2 or H_2 or $H_2/X = 3$ (in which X = N_2, CO_2 or CO), GHSV ≈ 16 000 $mL_{total\ flow}$ mL_{cat}^{-1} h^{-1}. Conversion variation was due to catalyst deactivation during runtime.

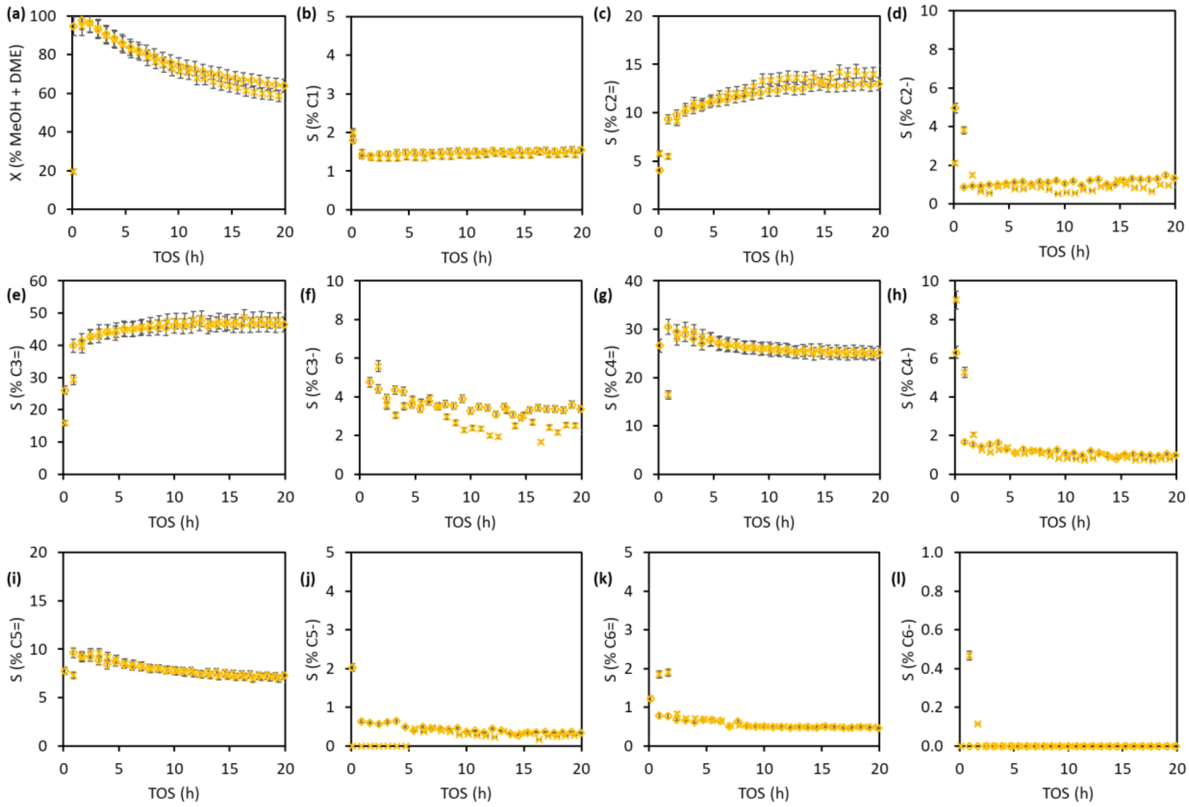

Supporting Figure 16. Reproducibility runs of CoAPO-18 with error bars at 95 % confidence interval. Reaction conditions: 350 °C, 20 bar, 1 bar MeOH (WHSV = 2.5 $g_{MeOH}\ g_{cat}^{-1}\ h^{-1}$), 0.6 bar Ar internal standard, 18.4 bar $H_2/CO = 3$, GHSV ≈ 16 000 $mL_{total\ flow}\ mL_{cat}^{-1}\ h^{-1}$.

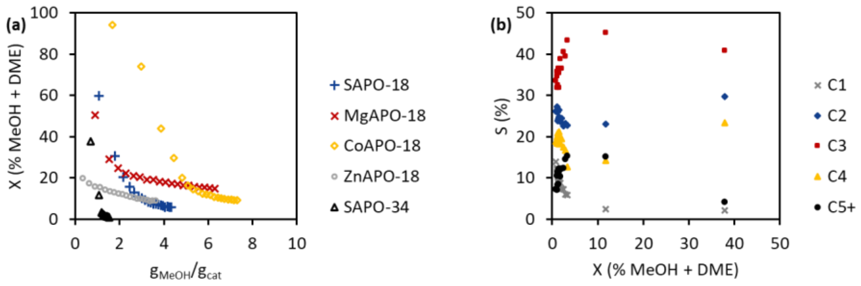

Supporting Figure 17. Catalytic performance of commercial SAPO-34 (from ACS Materials) at high pressure MTO reaction conditions. Reaction conditions: 350 °C, 20 bar, 1 bar MeOH (WHSV = 2.0 $g_{MeOH}\ g_{cat}^{-1}\ h^{-1}$), 0.6 bar Ar internal standard, 18.4 bar N_2, GHSV ≈ 14 000 $mL_{total\ flow}\ mL_{cat}^{-1}\ h^{-1}$. (a) Activity in terms % sum of MeOH and DME conversion over MeOH conversion capacity. The MAPO-18s were tested at identical conditions except with a higher WHSV (MeOH) = 2.5 $g_{MeOH}\ g_{cat}^{-1}\ h^{-1}$. (b) Selectivity vs. conversion of SAPO-34. Conversion variation was due to catalyst deactivation during runtime.

Supporting Table 3. Catalytic performance of MAPO-18s in various reaction feeds. Reaction conditions: 350 °C, 20 bar, 1 bar MeOH (WHSV = 2.5 g_{MeOH} g_{cat}^{-1} h^{-1}), 0.6 bar Ar internal standard, 18.4 bar N_2 or H_2 or $H_2/X = 3$ (in which X = N_2, CO_2 or CO), GHSV ≈ 16 000 $mL_{total\ flow}$ mL_{cat}^{-1} h^{-1}.

Catalyst	Reaction Conditions	Olefin-to-paraffin ratio			Ethylene/Propylene ratio	DME/MeOH ratio
		C2 =/-	C3 =/-	C4 =/-		
SAPO-18	N_2	39.2	53.6	27.2	0.38	5.4
	H_2	0.0	0.2	0.6	0.00	4.5
	$H_2/N_2 = 3$	0.3	1.0	0.9	0.09	5.0
	$H_2/CO_2 = 3$	0.4	1.8	1.9	0.12	5.0
	$H_2/CO = 3$	5.2	13.6	8.1	0.27	4.8
MgAPO-18	N_2	58.2	110.5	122.6	0.25	4.1
	H_2	0.0	0.1	0.6	0.00	21.6
	$H_2/N_2 = 3$	0.1	1.0	1.8	0.04	20.1
	$H_2/CO_2 = 3$	0.1	1.0	2.2	0.03	21.3
	$H_2/CO = 3$	16.4	19.4	38.0	0.20	19.0
CoAPO-18	N_2	58.1	97.3	78.6	0.36	5.1
	H_2	0.0	0.2	0.9	0.00	-
	$H_2/N_2 = 3$	0.3	1.7	2.7	0.09	21.6
	$H_2/CO_2 = 3$	0.1	1.2	2.3	0.05	18.5
	$H_2/CO = 3$	22.8	19.9	30.6	0.28	23.9
ZnAPO-18	N_2	57.3	115.1	101.2	0.29	0.5
	H_2	0.0	0.4	1.2	0.02	0.7
	$H_2/N_2 = 3$	0.5	0.9	1.0	0.20	0.7
	$H_2/CO_2 = 3$	0.4	1.5	2.0	0.15	0.8
	$H_2/CO = 3$	60.8	105.8	93.6	0.30	0.8

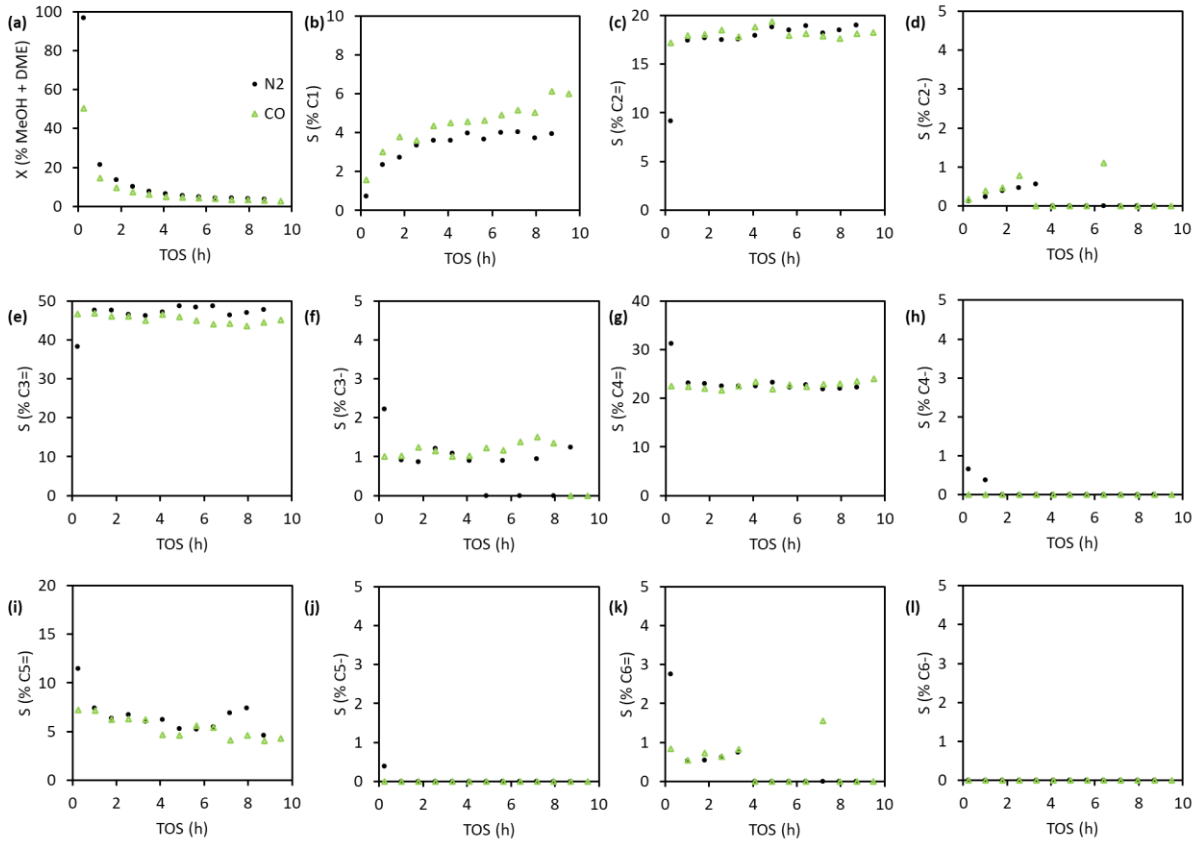

Supporting Figure 18. Catalytic performance of SAPO-18 in MeOH carbonylation reaction. Reaction conditions: 350 °C, 20 bar, 1 bar MeOH (WHSV = 2.5 $g_{MeOH}\ g_{cat}^{-1}\ h^{-1}$), 0.6 bar Ar internal standard, 18.4 bar N_2 or CO, GHSV ≈ 16 000 $mL_{total\ flow}\ mL_{cat}^{-1}\ h^{-1}$.

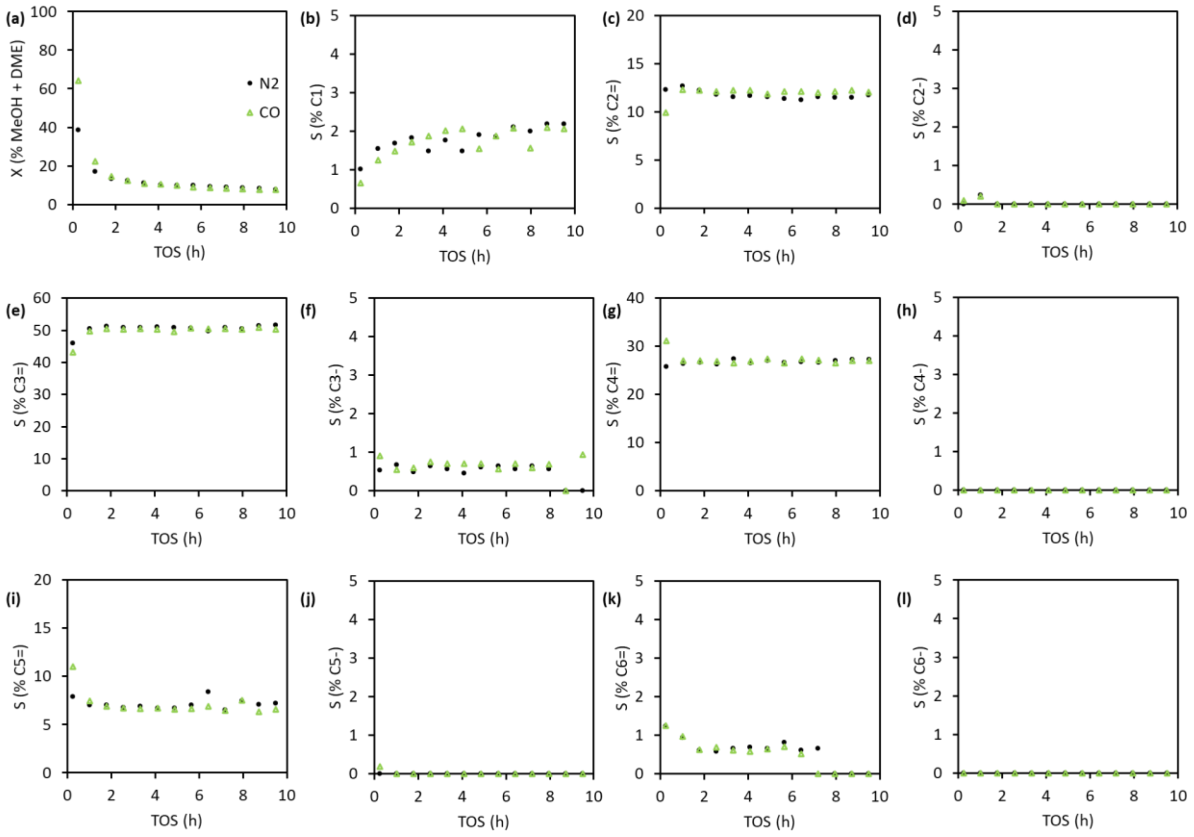

Supporting Figure 19. Catalytic performance of MgAPO-18 in MeOH carbonylation reaction. Reaction conditions: 350 °C, 20 bar, 1 bar MeOH (WHSV = 2.5 g_{MeOH} g_{cat}^{-1} h^{-1}), 0.6 bar Ar internal standard, 18.4 bar N_2 or CO, GHSV ≈ 16 000 $mL_{total\ flow}$ mL_{cat}^{-1} h^{-1}.

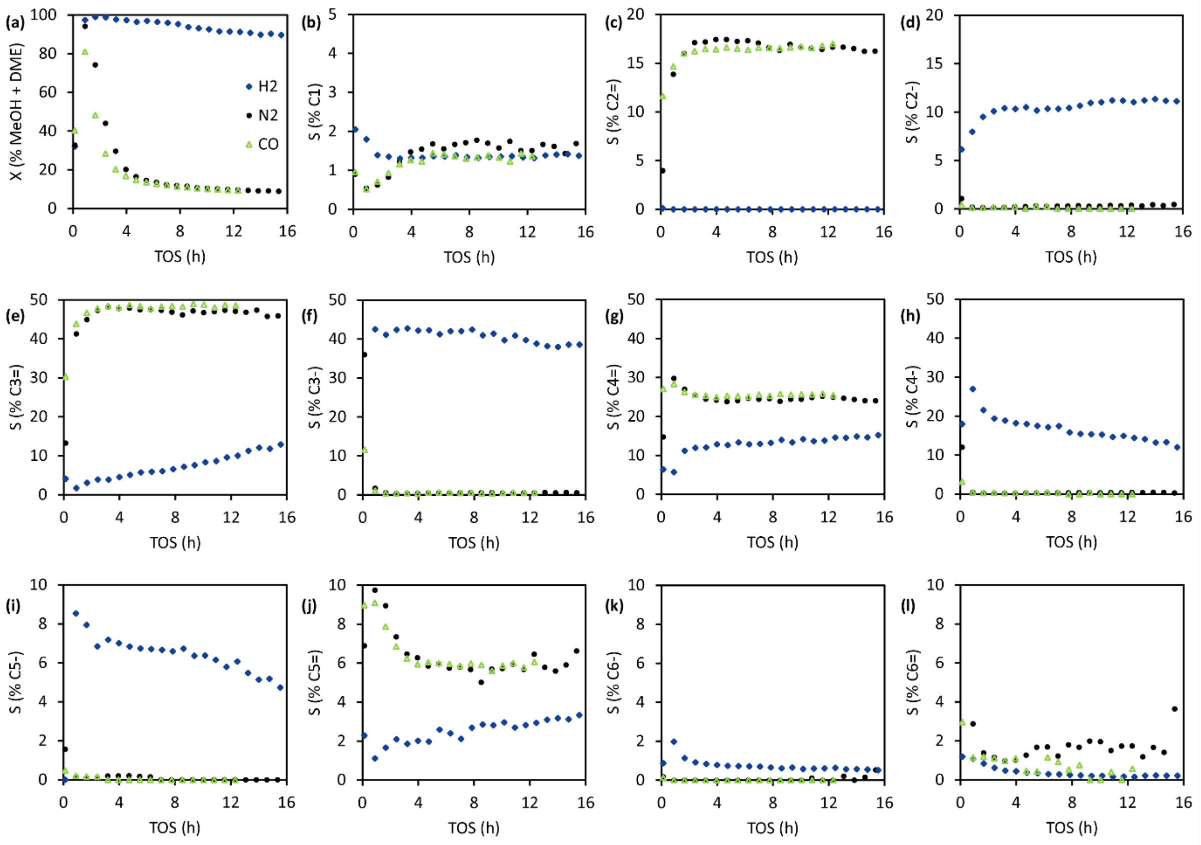

Supporting Figure 20. Catalytic performance of CoAPO-18 in MeOH carbonylation reaction. Reaction conditions: 350 °C, 20 bar, 1 bar MeOH (WHSV = 2.5 g_{MeOH} g_{cat}^{-1} h^{-1}), 0.6 bar Ar internal standard, 18.4 bar N_2 or H_2 or CO, GHSV ≈ 16 000 $mL_{total\ flow}$ mL_{cat}^{-1} h^{-1}.

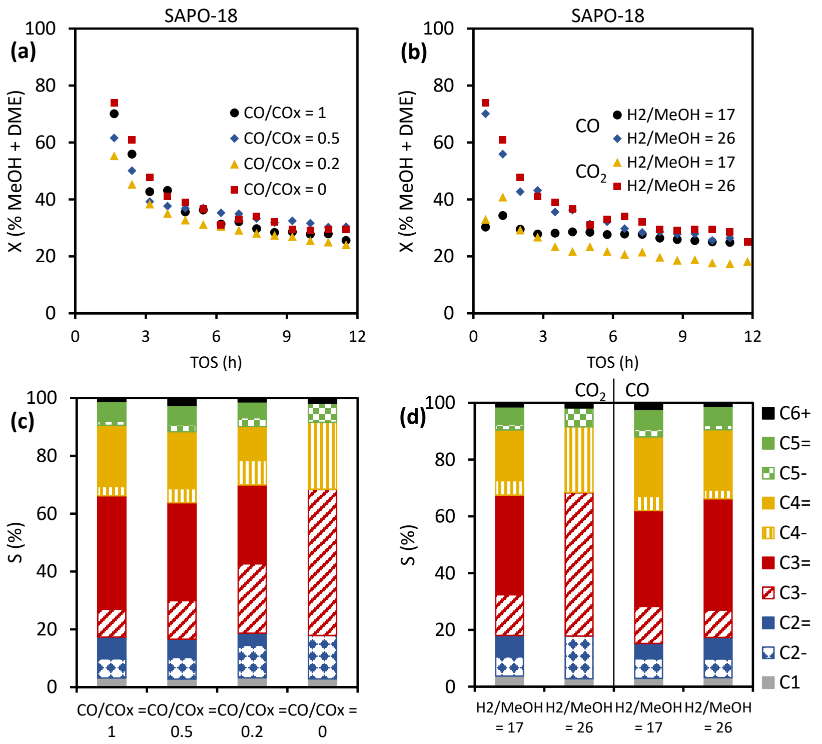

Supporting Figure 21. Effect of CO on the catalytic performance of SAPO-18s. Reaction conditions: 350 °C, 20 bar, 0.5 bar MeOH (WHSV = 2.5 g_{MeOH} g_{cat}^{-1} h^{-1}), 0.7 bar Ar internal standard, 18.8 bar H_2/CO_x = 3, GHSV ≈ 16 000 $mL_{total\ flow}$ mL_{cat}^{-1} h^{-1}. (a and b) Activity in terms % sum of MeOH and DME conversion over time-on-stream, and (c and d) Product selectivity at TOS = 10 h.

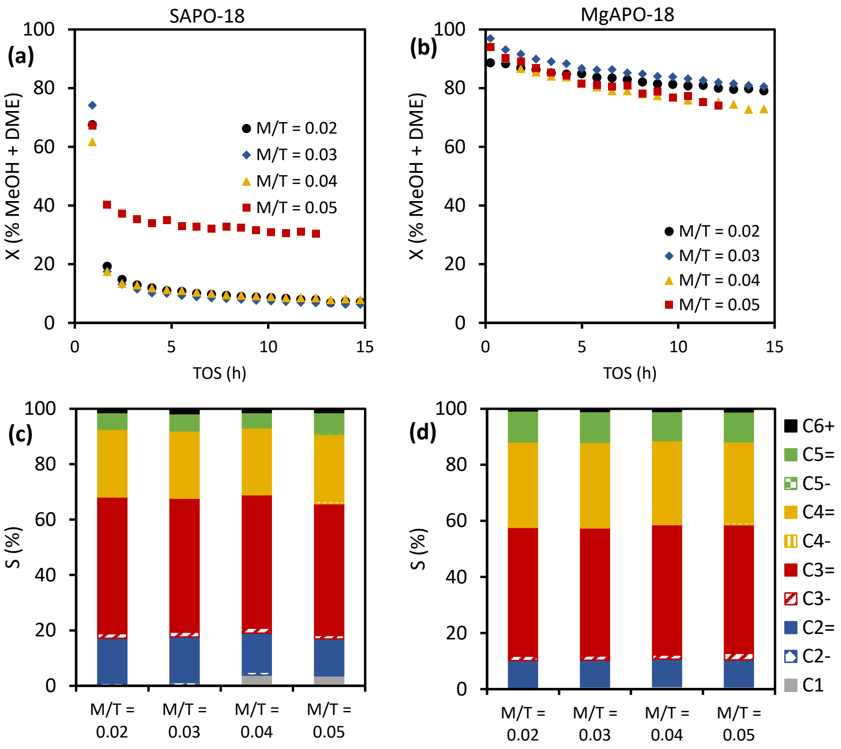

Supporting Figure 22. Effect of MAPO-18 M/T ratio on the catalytic performance of MAPO-18s. Reaction conditions: 350 °C, 20 bar, 1 bar MeOH (WHSV = 2.5 g_{MeOH} g_{cat}^{-1} h^{-1}), 0.6 bar Ar internal standard, 18.4 bar H_2/CO = 3, GHSV ≈ 16 000 $mL_{total\ flow}$ mL_{cat}^{-1} h^{-1}. a – b | Activity in terms % sum of MeOH and DME conversion over time-on-stream for (a) SAPO-18, (b) MgAPO-18. c – d | Product selectivity at TOS = 10 h for (c) SAPO-18, (d) MgAPO-18.

S4: Supporting DFT calculations

Optimized SAPO-18 lattice with ethene molecule

Final Coordinates Ethene in SAPO-18 (Angstroms)

Lattice:

translation vector [a0]	1	25.910790790	0.000000000	0.000000000
translation vector [a0]	2	0.000000000	24.059048160	0.000000000
translation vector [a0]	3	-0.006124912	0.000000000	35.093158470

Final Coordinates (Angstroms)

	ATOM	X	Y	Z
1	Al	1.463004	0.626289	3.034494
2	Al	1.500503	10.018531	17.569548
3	Al	10.540843	11.659187	0.886299
4	P	3.102360	11.569994	1.046574
5	P	12.177948	10.007021	17.422238
6	P	12.147021	0.486896	3.001458
7	O	11.151262	12.205783	2.427644
8	O	11.901423	11.070626	-0.065851
9	O	-0.136920	9.415814	17.641097
10	O	1.906715	10.725085	0.555199
11	O	2.675555	12.371926	2.277719
12	O	13.566884	0.128937	2.510969
13	O	11.804948	1.916828	2.550784
14	O	9.330346	10.440339	1.122897
15	O	1.545871	0.443031	4.758351
16	O	2.609140	8.741993	17.170499
17	O	12.102169	10.675570	16.037289
18	O	9.877172	0.280245	18.543214
19	Al	8.317358	6.832600	3.020084
20	Al	8.450832	3.677573	17.334300

21	Al	3.714182	5.305472	0.818205
22	P	9.958861	5.266278	0.918873
23	Si	5.338827	3.552416	17.440827
24	P	5.271536	6.765604	2.986099
25	O	4.276218	5.752733	2.420046
26	O	5.037739	4.795277	-0.177794
27	O	6.851318	3.075510	17.370974
28	O	8.736239	4.754184	0.134144
29	O	9.485884	5.764329	2.291135
30	O	6.692642	6.419704	2.489684
31	O	4.917114	8.192728	2.534038
32	O	2.450913	4.115642	1.003109
33	O	8.376161	6.719435	4.751499
34	O	9.642622	2.418891	17.278550
35	O	5.043066	4.312238	15.878208
36	O	3.063157	6.754313	18.602005
37	Al	12.181819	0.603652	6.236590
38	Al	12.148924	10.012373	10.270571
39	Al	3.104334	11.663916	8.379202
40	P	10.513226	11.600235	8.244081
41	P	1.471561	10.008179	10.422513
42	P	1.482418	0.483894	6.277779
43	O	2.495008	12.212392	6.839846
44	O	1.751960	11.044019	9.314504
45	O	13.790993	9.407590	10.197686
46	O	11.727093	10.799821	8.760355
47	O	10.938907	12.401192	7.014403
48	O	0.069978	0.103947	6.779890
49	O	1.813512	1.910850	6.750608
50	O	4.340273	10.476662	8.149109

51	O	12.059028	0.430265	4.523007
52	O	11.076683	8.684854	10.588761
53	O	1.531792	10.707732	11.790656
54	O	3.756528	0.278789	9.305959
55	Al	5.283448	6.887519	6.224279
56	Al	5.239198	3.690797	10.414966
57	Al	9.903816	5.264129	8.393070
58	P	3.691833	5.252205	8.335985
59	P	8.348524	3.615790	10.528185
60	P	8.323544	6.761196	6.272489
61	O	9.272925	5.689990	6.824794
62	O	8.595655	4.631992	9.393691
63	O	6.892861	3.133955	10.462124
64	O	4.948160	4.580169	8.936230
65	O	4.082685	5.893787	7.004753
66	O	6.897276	6.464138	6.787097
67	O	8.747370	8.157958	6.758142
68	O	11.186938	4.114777	8.234356
69	O	5.206199	6.704990	4.510708
70	O	4.132719	2.364010	10.602117
71	O	8.605901	4.308117	11.885959
72	O	10.500784	6.707272	9.191643
73	Al	12.143870	12.334579	15.458433
74	Al	12.126942	2.865054	1.106701
75	Al	3.038973	1.249774	17.709266
76	P	10.590762	1.274430	17.601906
77	P	1.488592	2.951478	1.198487
78	P	1.487113	12.523480	15.540299
79	O	2.424670	0.857715	16.112001
80	O	1.709687	1.855841	18.704576

81	O	13.761212	3.473048	1.071748
82	O	11.869766	1.802800	18.288986
83	O	10.975253	0.582598	16.290707
84	O	0.034751	13.022259	15.650367
85	O	1.638285	11.203903	16.301186
86	O	4.315260	2.355832	17.532397
87	O	11.764844	12.263657	13.763538
88	O	10.979427	4.156284	1.127072
89	O	1.697509	2.319555	2.592810
90	O	3.574403	12.491972	-0.089018
91	Al	5.352847	6.076051	15.540971
92	Al	5.257399	9.274451	1.196479
93	Al	9.928782	7.676993	17.702333
94	P	3.680172	7.745464	17.604960
95	P	8.362932	9.263233	1.215380
96	P	8.388595	6.099819	15.528626
97	O	9.393483	7.071697	16.153259
98	O	8.548755	8.315479	18.580860
99	O	6.926460	9.791063	1.194487
100	O	4.884055	8.468552	18.244926
101	O	4.158260	7.031126	16.327767
102	O	6.976484	6.437454	16.075499
103	O	8.728497	4.643060	15.867546
104	O	11.158299	8.879921	17.514008
105	O	5.266752	6.099671	13.806218
106	O	4.267721	10.674009	1.455926
107	O	8.636940	8.494490	2.521820
108	O	10.626955	6.404240	0.124795
109	Al	1.490278	12.359546	12.389132
110	Al	1.514954	2.880846	8.180425

111	Al	10.548394	1.249883	10.168557
112	P	3.101561	1.287713	10.267366
113	P	12.144568	2.937667	8.087893
114	P	12.140175	12.505661	12.305156
115	O	11.190259	0.833309	11.733514
116	O	11.852975	1.876195	9.170018
117	O	-0.132428	3.446782	8.268082
118	O	1.848925	1.897567	9.605298
119	O	2.685701	0.612255	11.579468
120	O	13.588542	13.018966	12.168178
121	O	11.990009	11.180311	11.550876
122	O	9.267637	2.408512	10.378033
123	O	1.882843	12.284249	14.079825
124	O	2.602525	4.220903	8.081086
125	O	11.975287	2.297822	6.698467
126	O	9.985003	12.531402	9.354860
127	Al	8.344324	6.010093	12.300013
128	Al	8.392782	9.262550	8.076393
129	Al	3.745514	7.694573	10.183145
130	P	9.956333	7.720237	10.214379
131	P	5.284311	9.284938	8.031741
132	P	5.300729	6.123984	12.277323
133	O	4.341185	7.199848	11.758583
134	O	5.074627	8.311913	9.213373
135	O	6.729143	9.786361	8.067756
136	O	8.753659	8.476054	9.609844
137	O	9.509307	7.008535	11.493153
138	O	6.719685	6.428117	11.755973
139	O	4.875071	4.727487	11.790594
140	O	2.512117	8.892529	10.385756

141	O	8.413244	6.278546	14.011500
142	O	9.384079	10.663369	7.829077
143	O	4.999068	8.559624	6.704785
144	O	3.135651	6.294100	9.323595
145	H	5.081185	3.670236	15.128976
146	C	5.664229	1.397165	14.687911
147	C	5.980854	2.209101	13.685664
148	H	4.781823	0.768456	14.644902
149	H	6.267499	1.331494	15.587635
150	H	5.368738	2.242206	12.792332
151	H	6.873881	2.830460	13.695857

Optimized MgAPO-18 lattice with ethene molecule

Final Coordinates Ethene in MgALPO-18 (Angstroms)

Lattice:

translation vector [a0] 1 25.910790790 0.000000000 0.000000000

translation vector [a0] 2 0.000000000 24.059048160 0.000000000

translation vector [a0] 3 -0.006124912 0.000000000 35.093158470

	ATOM	X	Y	Z
1	Al	1.409561	0.370744	3.081720
2	Al	1.571263	9.835621	17.432512
3	Al	10.602115	11.580913	0.820240
4	P	3.047490	11.398618	1.040635
5	P	12.110325	9.894053	17.378241
6	P	12.106387	0.402157	3.063203
7	O	11.042890	12.290986	2.351125
8	O	11.934036	10.637556	0.145806
9	O	-0.124302	9.537146	17.210321
10	O	1.886659	10.593203	0.416259

11	O	2.558011	12.035690	2.343189
12	O	13.495671	-0.021705	2.537733
13	O	11.899205	1.906349	2.820520
14	O	9.234692	10.527791	1.028244
15	O	1.469556	0.197414	4.803339
16	O	2.396998	8.318256	17.291345
17	O	11.624599	10.774876	16.212428
18	O	10.166694	0.086887	18.234751
19	Al	8.540739	6.975419	3.061319
20	Mg	8.349916	3.387782	17.818615
21	Al	3.853354	5.072668	0.977526
22	P	10.032259	5.242949	1.041808
23	P	5.254364	3.353624	17.370342
24	P	5.482809	6.664297	3.026322
25	O	4.588807	5.540399	2.490639
26	O	5.080699	4.456058	-0.115016
27	O	6.615238	2.720652	17.479971
28	O	8.698592	4.655640	0.629089
29	O	9.848874	6.146995	2.280580
30	O	6.946124	6.325001	2.700477
31	O	5.098892	8.009327	2.391664
32	O	2.589219	3.928143	1.288496
33	O	8.800789	6.840310	4.769394
34	O	9.936517	2.444383	17.388960
35	O	5.076586	4.046464	15.985340
36	O	3.189057	6.500159	18.773535
37	Al	12.131862	0.520011	6.253348
38	Al	12.142466	9.994230	10.384447
39	Al	3.042316	11.442887	8.398375
40	P	10.546685	11.593964	8.372726

41	P	1.457580	9.818757	10.536580
42	P	1.459830	0.282056	6.319912
43	O	2.341616	11.888117	6.870235
44	O	1.742292	10.828001	9.407810
45	O	13.738393	9.288394	10.363994
46	O	11.772045	10.798137	8.869427
47	O	10.918877	12.314661	7.079805
48	O	0.022989	0.115995	6.865263
49	O	1.999413	1.653083	6.767848
50	O	4.265365	10.245287	8.192686
51	O	11.992753	0.148170	4.569301
52	O	10.968594	8.743695	10.656924
53	O	1.593372	10.541351	11.888332
54	O	3.692904	0.132141	9.191988
55	Al	5.497454	6.730472	6.248331
56	Al	5.331704	3.503844	10.477109
57	Al	10.091133	5.296652	8.449046
58	P	3.791011	5.134798	8.288400
59	P	8.418484	3.588388	10.382875
60	P	8.526861	6.891076	6.262343
61	O	9.616431	6.090734	6.983447
62	O	8.699045	4.457292	9.142925
63	O	7.003082	3.007201	10.289450
64	O	4.935598	4.636354	9.199981
65	O	4.393813	5.581193	6.955996
66	O	7.155264	6.276589	6.617317
67	O	8.554152	8.366125	6.694756
68	O	11.393332	4.199217	8.123465
69	O	5.270335	6.786679	4.535619
70	O	4.249632	2.154834	10.513629

71	O	8.557333	4.440056	11.664415
72	O	10.625215	6.478295	9.639521
73	Al	12.184512	12.258939	15.470067
74	Al	12.199021	2.874662	1.370834
75	Al	2.909719	1.151891	17.780624
76	P	10.902814	1.316905	17.638590
77	P	1.531324	2.835243	1.383052
78	P	1.735352	12.185934	15.501809
79	O	2.257879	0.691101	16.236219
80	O	1.639311	1.849500	18.769248
81	O	13.848516	3.472051	1.364526
82	O	12.030972	1.729281	18.616603
83	O	11.562943	0.912587	16.302246
84	O	0.205849	12.215638	15.499019
85	O	2.211654	10.898306	16.191290
86	O	4.157382	2.306265	17.543634
87	O	11.674945	12.367171	13.808290
88	O	11.072358	4.172894	1.396391
89	O	1.739737	2.050558	2.694260
90	O	3.527866	12.475529	0.054165
91	Al	5.211983	5.733938	15.580385
92	Al	5.262831	9.135122	1.069540
93	Al	9.996302	7.513453	17.675932
94	P	3.610753	7.471140	17.659810
95	P	8.358793	9.286664	1.147919
96	P	8.280545	6.028182	15.544808
97	O	9.250927	7.028309	16.137204
98	O	8.736455	8.230538	18.655464
99	O	6.903418	9.702286	0.914845
100	O	4.784528	8.347903	18.141557

101	O	4.050334	6.714043	16.403332
102	O	6.858789	6.289015	16.017048
103	O	8.660513	4.556211	16.088473
104	O	11.291136	8.605688	17.344586
105	O	5.029913	6.017321	13.872352
106	O	4.223262	10.488730	1.381209
107	O	8.520793	8.664738	2.547169
108	O	10.633122	6.095227	-0.112346
109	Al	1.640403	12.208851	12.445320
110	Al	1.639796	2.730194	8.104816
111	Al	10.714892	1.316721	10.196294
112	P	3.139677	1.148385	10.206835
113	P	12.236001	2.934880	7.976558
114	P	12.233210	12.504464	12.390785
115	O	11.469048	0.909629	11.706004
116	O	11.956147	1.962454	9.141394
117	O	0.005285	3.328707	8.021153
118	O	1.901560	1.857692	9.616314
119	O	2.733681	0.449077	11.502395
120	O	13.729153	12.868904	12.405688
121	O	12.028631	11.173557	11.658649
122	O	9.427983	2.447098	10.481220
123	O	2.267381	12.208006	14.067873
124	O	2.778197	4.032729	8.016391
125	O	11.898882	2.232710	6.645563
126	O	10.094574	12.596474	9.449852
127	Al	8.218045	6.036781	12.299754
128	Al	8.361206	9.268644	8.175778
129	Al	3.660603	7.465191	10.172560
130	P	9.945256	7.657370	10.359877

131	P	5.309220	9.133630	8.230492
132	P	5.211310	5.880996	12.365559
133	O	4.095012	6.649957	11.653873
134	O	5.075943	8.226182	9.457758
135	O	6.700283	9.763242	8.357361
136	O	8.787966	8.188506	9.494100
137	O	9.364303	7.189460	11.702470
138	O	6.570953	6.485920	11.933757
139	O	5.185890	4.386573	11.996019
140	O	2.424966	8.641102	10.491012
141	O	8.432269	5.918948	14.038241
142	O	9.372248	10.663101	8.096309
143	O	5.193434	8.319074	6.934174
144	O	3.075587	6.306673	8.988605
145	H	22.863202	16.729328	15.421027
146	C	10.306364	15.646491	13.921076
147	C	9.148590	15.020300	14.096264
148	H	11.181195	15.386351	14.505559
149	H	10.426272	16.412754	13.162954
150	H	9.039036	14.230759	14.832082
151	H	8.268846	15.264179	13.507129

S5: Supporting references

1. Olsbye, U. *et al.* Conversion of methanol to hydrocarbons: How zeolite cavity and pore size controls product selectivity. *Angew. Chemie Int. Ed.* **51**, 5810–5831 (2012).

2. Sławiński, W. A., Wragg, D. S., Akporiaye, D., & Fjellvåg, H. Intergrowth structure modelling in silicoaluminophosphate SAPO-18/34 family. *Micropor. Mesopor. Mater.,* **195**, 311-318 (2014).

3. Simmen, A., McCusker, L.B., Baerlocher, Ch. & Meier, W.M. The structure determination and rietveld refinement of the aluminophosphate AIPO4-18. *Zeolites*, **11**, 654-661 (1991).

4. Sun, C., Wang, Y., Chen, H., Wang, X., Wang, C. & Zhang, X. Seed-assisted synthesis of hierarchical SAPO-18/34 intergrowth and SAPO-34 zeolites and their catalytic performance for the methanol-to-olefin reaction. *Catal. Today* **355**, 188-198 (2020).

5. Frache, A., Gianotti, E. & Marchese, L. Spectroscopic characterisation of microporous aluminophosphate materials with potential application in environmental catalysis. *Catal. Today* **77**, 371–384 (2003).

6. Gianotti, E., Vishnuvarthan, M., Berlier, G., Marchese, L. & Coluccia, S. FTIR study of cobalt containing aluminophosphates with chabasite like structure by using CO and NO as molecular probes. *Catal. Letters* **133**, 27–32 (2009).

7. Potter, M. E. Down the Microporous Rabbit Hole of Silicoaluminophosphates: Recent Developments on Synthesis, Characterization, and Catalytic Applications. *ACS Catal.* **10**, 9758–9789 (2020).

8. Chen, J. *et al.* SAPO-18 Catalysts and Their Brønsted Acid Sites. *J. Phys. Chem.* **98**, 10216–10224 (1994).
